# Targeting the ac4C ‘Writer’ NAT10 enhances pancreatic cancer immunotherapy via dual modulation of CD8+ T cells and tumor cells

**DOI:** 10.1038/s41419-025-08156-0

**Published:** 2025-11-07

**Authors:** Leisheng Wang, Enhong Chen, Shuo Zhang, Wen Xiang, Hao Ye, Yifei Fu, Shiwei Xu, Qin Wang, Yue Tao, Dongjie Yang, Youzhao He, Shudong Hu, Yong Mao, Hao Hu

**Affiliations:** 1 Affiliated Hospital of Jiangnan University, Wuxi, China; 2 Wuxi Medical College, Jiangnan University, Wuxi, China; 3 Wuxi Ninth People’s Hospital, Wuxi, China

**Keywords:** Pancreatic cancer, Epigenetics

## Abstract

Pancreatic cancer (PC) is highly lethal because of its immunosuppressive tumor microenvironment and resistance to immunotherapy. This study explored the role of NAT10-mediated N4-acetylcytidine (ac4C) RNA modification in pancreatic cancer progression and immune evasion. NAT10 (N-acetyltransferase 10) is overexpressed in pancreatic cancer tissues and correlates with poor prognosis. Mechanistically, NAT10 stabilizes ETS2 mRNA through ac4C acetylation, forming a positive feedback loop that upregulates NAT10 and PD-L1, thereby suppressing CD8 + T cell infiltration and promoting immune evasion. In addition, NAT10 stabilizes KRT8 mRNA via ac4C acetylation, which drives cancer cell proliferation and metastasis. Single-cell RNA sequencing analysis revealed enhanced interactions between pancreatic cancer epithelial cells with high NAT10 and KRT8 expression, and T cells, thereby providing new insights into the immune microenvironment. In vivo, NAT10 knockdown significantly inhibited tumor growth, enhanced CD8 + T cell infiltration, and reduced lung metastasis. Notably, combination therapy with an NAT10 inhibitor and anti-PD-L1 antibody demonstrated superior antitumor efficacy compared to monotherapy. In conclusion, NAT10 promotes pancreatic cancer progression and immune evasion by regulating the ETS2-PD-L1 axis and stabilizing KRT8 mRNA, highlighting its potential as a therapeutic target for overcoming immunotherapy resistance.

## Highlights

- NAT10 Promotes Pancreatic Cancer Immune Suppression via ac4C-Mediated Regulation of the ETS2-PD-L1 Axis and KRT8.
- NAT10-ETS2 Positive Feedback Loop Amplifies Malignant Progression and Immune Suppression.
- Combination Therapy Targeting NAT10 and PD-L1 is More Effective Than Monotherapy.

## Introduction

Pancreatic cancer (PC) is one of the most lethal malignancies, with a steadily increasing incidence and a five-year survival rate of approximately 13% [1, 2]. This increasing burden poses a significant threat to public health. Despite advances in treatments, such as surgery, adjuvant and neoadjuvant chemoradiotherapy, and targeted therapies, these approaches have not significantly improved patient outcomes[3]. Immunotherapy, particularly immune checkpoint blockade (ICB) targeting PD-1/PD-L1, has shown remarkable survival benefits in other cancers, and has become a major focus of cancer research. However, the inherent “cold tumor” phenotype of PC, coupled with its highly immunosuppressive tumor microenvironment (TME), results in high resistance to immunotherapy [4, 5]. These challenges underscore the urgent need to investigate the mechanisms driving the immunosuppressive TME in PC, identify key mediators of immunotherapy resistance, and develop novel strategies for combination therapies to improve patient prognosis [6, 7].

N4-acetylcytidine (ac4C) is a highly conserved RNA modification present in both eukaryotes and prokaryotes [8, 9], which plays essential roles in promoting protein translation, stabilizing RNA, facilitating alternative splicing, and regulating gene expression [10, 11]. In addition, ac4C plays important roles in tumor malignant progression, including bladder cancer [12], gastric cancer [13], and colorectal cancer [14]. NAT10, a member of the GCN5-related N-terminal acetyltransferase (NAT) family, is the only identified “writer” of ac4C modifications and modifies various RNA types, including rRNA, tRNA, and mRNA [15]. Aberrant NAT10 overexpression has been linked to increased ac4C acetylation, upregulation of downstream gene expression, and promotion of tumorigenesis and progression in multiple cancers [16]. A previous study has demonstrated that NAT10 promotes tumor malignant progression in prostate cancer [11]. In PC, NAT10 promotes tumor metastasis via ac4C-mediated modifications on receptor tyrosine kinases [17]. In recent years, several studies have indicated that NAT10 regulates PD-L1 expression and facilitates immune suppression in multiple cancers, including breast [18], colorectal [19], and cervical cancers [20]. These findings suggest that targeting NAT10 or combining it with ICB represents a promising strategy to overcome the immunosuppressive tumor microenvironment.

The limited efficacy of immunotherapy in PC is largely attributed to its acquired immune privilege, often described as a “cold tumor” phenotype [21]. This immune-resistant state arises from a combination of factors, including an immunosuppressive TME, stromal heterogeneity, insufficient immune cell infiltration, and low tumor mutational burden [22]. To overcome these barriers, combination therapies that enhance immune co-stimulation are being explored to increase the efficacy of immunotherapy in patients with pancreatic ductal adenocarcinoma (PDAC) [23]. Preliminary research suggests that NAT10 enhances gene expression by stabilizing RNA and regulating biological processes, such as the tumor cell cycle, ferroptosis, and metabolic pathways, all of which drive tumor progression [15]. Emerging evidence also implicates aberrant NAT10 expression in tumor immune evasion, although systematic in vivo and in vitro studies on its precise mechanisms remain limited [24]. We hypothesized that targeting NAT10 could improve immune signatures, attenuate the immunosuppressive TME, and enhance the antitumor effects of PD-L1 blockade therapy in PDAC.

This study investigated the role and mechanism of action of NAT10 in PDAC progression, and its impact on patient prognosis. Specifically, we examined whether immune regulation is a critical mechanism underlying the effects of NAT10 and evaluated the therapeutic potential of combining NAT10 inhibition with PD-L1 blockade to overcome immune resistance and improve treatment outcomes.

## Materials and methods

### Clinical tissue samples

In this study, a total of 20 pairs of pancreatic cancer tissues and adjacent normal tissues were obtained from patients who underwent surgical treatment at the Affiliated Hospital of Jiangnan University, Wuxi, China. Fresh tissues were stored at −80 °C for subsequent protein and RNA extraction. This study was approved by the Ethics Committee of Jiangnan University Hospital, and informed consent was obtained from all patients prior to their participation (ethics number: LS2024065).

### Cell culture

hTERT-HPNE, SW 1990, PANC-1, Panc02, AsPC-1, BxPC-3, MIA PaCa-2, and CFPAC-1 cell lines were obtained from Zhongqiao Xinzhou (Shanghai, China). The hTERT-HPNE, SW 1990, PANC-1, and Panc02 cell lines were cultured in DMEM (Zhongqiao Xinzhou) supplemented with 10% FBS (Newzerum) and 1% PS (Proteinbio). AsPC-1 and BxPC-3 cells were cultured in RPMI-1640 medium (Zhongqiao Xinzhou), whereas CFPAC-1 cells were cultured in IMDM (Zhongqiao Xinzhou), supplemented with 10% fetal bovine serum and 1% PS. The MIA PaCa-2 cell line was cultured in DMEM (Zhongqiao Xinzhou) supplemented with 10% FBS, 1% PS, 2.5% horse serum, and 100 mM sodium pyruvate. All the cell lines were incubated at 37 °C in a 5% CO₂ atmosphere. All the cell lines were characterized by STR analysis and were free of mycoplasma contamination.

### Plasmid construction, lentivirus production, and cell transduction

Small interfering RNAs (siRNAs) specific for ETS2 and KRT8, along with their corresponding negative controls, were chemically synthesized by Sangon Biotech (Shanghai, China). Lipofectamine 2000 (Thermo Fisher Scientific) was used to facilitate the transfection of recombinant plasmids into pancreatic cancer cells following the detailed protocol provided by the manufacturer. Lentiviruses packaging shNAT10 and shNC were synthesized by GeneChem (Shanghai, China). The pancreatic cancer cell lines were treated with lentiviral particles and incubated for 48 h. Stable cell clones were established using puromycin selection. The ETS2 and KRT8 overexpression plasmids were purchased from GeneChem (Shanghai, China). Details of the plasmids, siRNAs, and shRNAs used are provided in Table S1 (Supporting Information).

### Animal studies

Animal experiments were approved by the Animal Ethics Committee of Jiangnan University (JN.No. 20231230c0320530[625]).C57BL/6 and BALB/c nude mice, aged 6–8 weeks, were purchased from Shanghai Southern Model Biotechnology Co., Ltd. and maintained under pathogen-free conditions.The males were chosen for the indicated experiments.All animals were randomly allocated.

For the xenograft model, PANC-1 or MIA PaCa-2 cells (1 × 10^6^ cells) suspended in 100 μL of phosphate-buffered saline (PBS) were injected subcutaneously into the armpits of BALB/c nude mice. Twenty-seven days post-injection, the mice were sacrificed, and the tumors were excised for weight measurement.

For the metastasis model, PANC-1 cells (1 × 10^6^ cells) suspended in 100 μL of PBS were injected into the tail vein of BALB/c nude. After 27 days, the mice were sacrificed, and their lung tissues were dissected to observe lung metastasis via HE staining.

To investigate the immune environment, mouse-derived pancreatic cancer cells (Panc02) stably transfected with sh-Nat10 or sh-NC were constructed, which were then injected subcutaneously into the armpits of C57BL/6 mice (1 × 10^6^ cells in 100 μLof PBS). After 27 days, the mice were sacrificed, and the tumors were removed to assess changes in immune cell populations.

For the combination therapy model, Panc02 cells (1 × 10^6^ cells) suspended in 100 μL of PBS were injected subcutaneously into the axilla of C57BL/6 mice, along with anti-PD-L1 (10 mg/kg) administered three times a week and remodelin (10 mg/kg). Thirty days after injection, the mice were sacrificed, and the tumors were excised to evaluate changes in the immune cells.

### Immunohistochemistry

Immunohistochemistry (IHC) assays were performed according to the specified protocol. Briefly, paraffin-embedded sections were dewaxed and rehydrated. For high-temperature antigen retrieval, the sections were treated with water after deparaffinization and subsequently incubated with 2% EDTA solution. Endogenous peroxidase activity was inhibited by the addition of 3% hydrogen peroxide. The sections were incubated with primary antibodies NAT10 (1:500, T510105, Abmart, China) and CD8 (1:500, 29896-1-AP, Proteintech, China) at 4 °C overnight. The following day, the sections were incubated with the secondary antibody at room temperature for one hour. The sections were stained and photographed. IHC staining results were quantified by multiplying the proportion of immunoreactive cells (81–100% = 4, 51–80% = 3, 11–50% = 2, 1–10% = 1, 0% = 0) by the staining intensity (strong = 3, medium = 2, weak = 1, negative = 0). The final ratings were categorized as strongly positive (3+; 9–12), moderately positive (2+; 6--8), weakly positive (1+; 2--4), or negative (0; 0--1).The antibodies used in this study are listed in the Supplementary Table S2.

### Multiplex Immunohistochemistry(mIHC)

Multiplex immunohistochemistry was conducted in a manner similar to that used for traditional immunohistochemistry. Briefly, paraffin-embedded sections were dewaxed, rehydrated, and antigen retrieval. Endogenous peroxidase activity was suppressed by exposure to 3% hydrogen peroxide. After blocking, the sections were incubated with multiple primary antibodies (NAT10 1:200, CD8 1:200, PD-L1 1:200) followed by incubation with horseradish peroxidase-conjugated secondary antibodies and tyramide signal amplification. The sections were subjected to microwave heat treatment after each tyramide signal amplification. Nuclei were stained with DAPI after all antigens were labeled. The antibodies used in this study are listed in the Supplementary Table S2.

### Flow cytometry

Cell surface markers were evaluated by flow cytometric analysis. Briefly, the tissue sample was converted into a cell suspension using a dissociating reagent, followed by washing the cells twice with cold PBS. Cells were first incubated with Fc blocking agent at room temperature in the dark for 15 min. Then, live or dead dye and cell surface fluorescent antibody were stained at room temperature in the dark for 15 min. All cells were acquired using a flow cytometer within 3 h. Data were evaluated using the FlowJo software. The antibodies used in this study are listed in the Supplementary Table S2.

### Western blot analysis

Total protein was extracted using RIPA cell lysis buffer (ProteinBio, China). The protein extracts were separated using 10% sodium dodecyl sulfate-polyacrylamide gel electrophoresis (SDS-PAGE), and then transferred onto polyvinylidene fluoride (PVDF) membranes. After blocking, the membrane was incubated with primary antibodies, followed by secondary antibodies. Protein expression was visualized using an enhanced chemiluminescence (ECL) developer (Millipore, USA). The antibodies used in this study are listed in the Supplementary Table S2.

### Quantitative Real-time PCR (qPCR)

Total RNA was extracted using TRIzol reagent (R401, Vazyme). Complementary DNA was acquired using the HiScript II Q Select RT SuperMix (#R23301). SYBR qPCR Master Mix (Q511‒02, Vazyme) was used in the ABI Prism 7500 system to measure the mRNA expression. GAPDH was used to normalize the RNA levels using the 2^-ΔΔCt^ method. For detailed primer sequences, refer to Table S1.

### Cell proliferation assay

Cell proliferation was measured using the CCK-8 Cell Counting Kit (Vazyme, China). Briefly, 100 μL of the cell suspension was added to each well of a 96-well plate. The wells were subsequently incubated for a specified duration before 10 μL CCK-8 solution was added. The mixture was incubated for an additional two hours. The optical density (OD) of each well was measured at 450 nm using a microplate reader.

### Transwell assays

The upper chamber (Corning, USA) was used to culture pancreatic cancer cells suspended in serum-free medium at a concentration of 5 × 10^4^ cells per well. In contrast, the lower chamber contained a medium supplemented with 10% FBS. The chambers were subsequently incubated for 24–72 h. After this period, the uncrossed cells in the upper layer were removed and the remaining cells were fixed with a fixative and stained. Finally, quantitative results of cell migration were obtained through microscopic observation and image analysis.

### mRNA stability assay

Pancreatic cancer cells were seeded in 6-well plates at 50% confluence and incubated for 24 h. The cells were then treated with 5 μg/mL actinomycin D (#HY-17559, MedChemExpress) and harvested at 0, 2, 4, 6, and 8 h for RNA extraction. RT‒qPCR was subsequently conducted to assess the mRNA half-life.

### Dual-luciferase reporter assay

Plasmids encoding NAT10 with a mutated ETS2 binding site and PD-L1 with a mutated ETS2 binding site, and the corresponding wild-type (WT) NAT10, and WT PD-L1 were constructed. Luciferase reporter gene assay was conducted according to the protocol outlined in the Dual Luciferase Reporter Assay Kit (DL101-01). The activities of firefly luciferase were measured in both the ETS2 knockdown and control groups, normalized to Renilla fluorescence. Additionally, Plasmids encoding KRT8 with ac4C acetylation site mutants and ETS2 with ac4C acetylation site mutants, and the corresponding WT KRT8 and WT ETS2 were constructed. The activities of firefly luciferase were measured in both the NAT10 knockdown and control groups, normalized to Renilla fluorescence.

### RNA sequencing (RNA‑seq) and acRIP sequencing (acRIP‑seq)

High-throughput RNA sequencing and acRIP sequencing were performed to determine the ac4C modifications of individual genes in pancreatic cancer cells. All data analyses and processing were performed by Epibiology (Guangzhou, China).

### RIP-qPCR and acRIP-qPCR

A Magna RIP RNA-binding protein immunoprecipitation kit (Millipore, MA, USA) was used for RIP experiments in accordance with the manufacturer’s instructions. Briefly, the cells were harvested and lysed using RIP lysis buffer. The magnetic beads were then individually treated with NAT10 antibody and anti-rabbit IgG (Millipore, Germany) for one–two hours at room temperature before the addition of cell lysates, which comprised approximately 2 × 10^7^ cells/sample. After washing, the beads were incubated with cell lysate for three hours at 4 °C. Following the collection and washing of the beads, phenol-chloroform extraction was performed to isolate RNA. Quantitative polymerase chain reaction (qPCR) was performed on the RNA-rich regions. We enhanced the methodology of the Magna meriptmm6A kit (17--10,499, Millipore) for acRIP analysis of NAT10 knockdown by replacing the m6A antibody with the ac4C antibody (ab252215, Abcam). The complete procedure was performed in accordance with the manufacturer’s instructions.

### ChIP‒qPCR

Chromatin immunoprecipitation (ChIP) detection was performed using a ChIP detection kit (#P2078, Beyotime, Shanghai, China) according to the manufacturer’s instructions. The cells were cross-linked with formaldehyde and sonicated to obtain an average fragment length of 200–1000 bp. Sheared chromatin was immunoprecipitated overnight at 4 °C using an anti-ETS2 antibody (#GTX116011, GeneTex), with IgG (Thermo Fisher, 10003D) serving as a negative control. The precipitated DNA was amplified using RT-PCR.

### Assay for Transposase-accessible Chromatin followed by sequencing

Briefly, sh-NC and sh-NAT10 PANC-1 cells (5 × 10^5^ cells) were treated as previously described. The final sample was purified and sequenced using an Illumina NovaSeq 6000 system. A sequencing service was provided by Guangzhou Epibiology [25].

### Single-cell data analysis

Single-cell datasets of pancreatic cancer, including GSE194247, GSE197177, GSE212966, and GSE235449, were downloaded from the GEO database, which provides single-cell data for 30 pancreatic cancer tissues and seven para-cancerous tissues. For data processing, dplyr 1.1.1 was utilized for data preprocessing and Seurat 4.3.0 for dimensionality processing. SeuratObject 4.1.3 was used for data storage and management. In terms of visualization, Graphics 4.2.2, and ggplot2 3.4.4 were used for plotting, UMAP was implemented via Seurat. Iggraph 2.0.3 was applied to construct and analyze the networks. Inferred communication between cell types or clusters was represented as a network, with nodes corresponding to cell groups and edges representing ligand–ligand-receptor-mediated interactions. CellChat was applied to explore intercellular ligand–receptor signaling, while NAT10 and KRT8 expression levels were determined through differential expression analysis and visualization.

### Statistical analysis

GraphPad Prism 9.0 was used for the statistical analysis of the data, which are presented as the means ± standard deviations. One-way ANOVA was used for multiple comparisons, whereas a two-tailed Student’s *t*-test was used for comparisons between two groups. The survival rate of PAAD patients was calculated via Kaplan‒Meier curves. Significant differences were defined as *P* < 0.05 (*), *P* < 0.01 (**), and *P* < 0.001 (***).

## Results

### NAT10 is highly expressed in pancreatic cancer and is associated with poor clinical prognosis

**Fig. 1:**
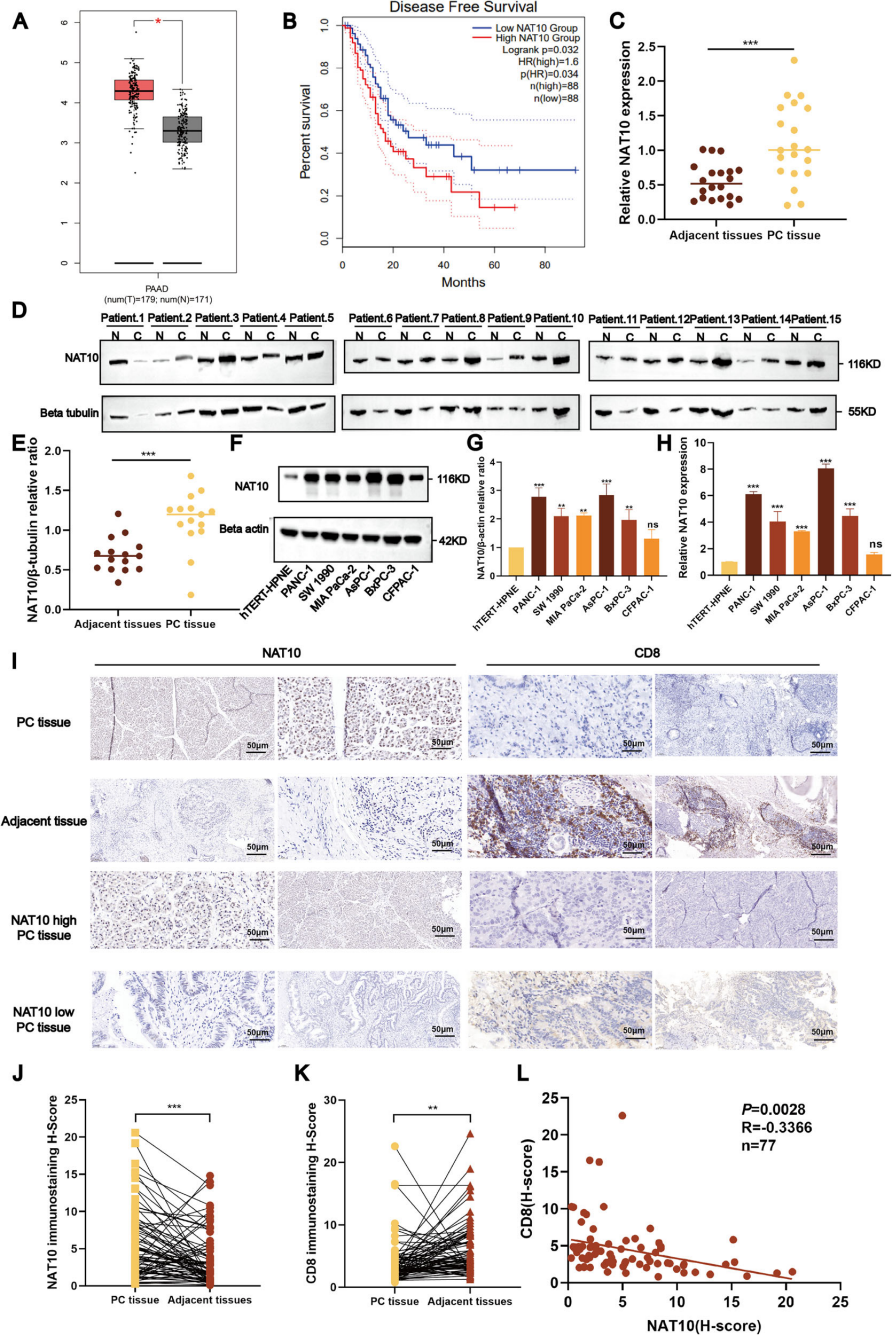
NAT10 is highly expressed in pancreatic cancer and inhibits immune cell infiltration. **A** NAT10 expression is significantly upregulated in pancreatic cancer tissues (GEPIA database). **B** High NAT10 expression is correlated with poor disease-free survival in pancreatic cancer patients (GEPIA database). **C** NAT10 mRNA levels are significantly elevated in pancreatic cancer tissues compared with adjacent normal tissues (*P* < 0.001). **D**, **E** Western blot analysis showing elevated NAT10 protein expression in pancreatic cancer tissues compared with adjacent normal tissues. **F**, **G** NAT10 protein levels are increased in pancreatic cancer cell lines compared with normal pancreatic ductal cells (hTERT-HPNE) (*P* < 0.001). **H** NAT10 mRNA levels are significantly elevated in pancreatic cancer cell lines (*P* < 0.001). **I** Immunohistochemistry (IHC) analysis revealed that NAT10 expression is localized to the nuclei of pancreatic cancer cells. **J** Quantitative analysis of the IHC results revealed significantly higher NAT10 H-scores in pancreatic cancer tissues (*P* < 0.001). **K** Quantitative analysis revealed significantly lower CD8 + T-cell infiltration in pancreatic cancer tissues (*P* < 0.01). **L** There was a negative correlation between NAT10 expression (Hscore) and CD8 + T-cell infiltration (R = -0.3366, *P* = 0.0028).

To explore the role of NAT10 in pancreatic cancer, analysis using the GEPIA database revealed significantly elevated NAT10 expression in pancreatic cancer tissues compared with that in normal tissues (Fig. 1A). Additionally, higher NAT10 expression was correlated with worse disease-free survival, suggesting its potential as a prognostic marker (Fig. 1B). To validate these findings, qPCR analysis of 20 paired pancreatic cancer and adjacent normal tissues confirmed that NAT10 expression was significantly increased in the tumor tissues (Fig. 1C). Western blot analysis of 15 paired samples further supported these results, showing consistently elevated NAT10 protein levels in cancerous tissues (Fig. 1D, E). Furthermore, NAT10 expression was evaluated in pancreatic cancer cell lines (BxPC-3, SW 1990, MIA PaCa-2, AsPC-1, PANC-1, and CFPAC-1) and the normal pancreatic ductal epithelial cell line, hTERT-HPNE. Both NAT10 protein (Fig. 1F, G) and mRNA (Fig. 1H) levels were significantly increased in cancer cell lines. Collectively, these results indicate that NAT10 is overexpressed in pancreatic cancer and is associated with a poor prognosis.

### NAT10 inhibits CD8 + T-cell infiltration in pancreatic cancer and is correlated with clinicopathological characteristics

Immunohistochemistry (IHC) analysis of samples from 77 patients with pancreatic cancer revealed that the NAT10 protein was predominantly localized in tumor cell nuclei and was significantly elevated in cancerous tissues compared to adjacent normal tissues (Fig. 1I, left panels). In contrast, CD8 + T cell infiltration was markedly reduced in the cancerous tissues (Fig. 1I, right panels). Quantitative IHC analysis revealed higher NAT10 H-scores in tumor tissues (Fig. 1J) and lower CD8+ T-cell H-scores (Fig. 1K). Further correlation analysis of 77 tissue samples revealed a negative association between NAT10 expression and CD8 + T cell infiltration (Fig. 1L). Clinical correlation studies also revealed that elevated NAT10 expression was significantly associated with unfavorable clinicopathological characteristics (Table 1). These findings suggest that NAT10 contributes to pancreatic cancer progression by inhibiting CD8+ T cell infiltration and promoting an immunosuppressive TME.

**Table 1:** Relationships between NAT10 expression and clinicopathological characteristics in 77 patients with pancreatic cancer.

Clinical parameter	Total [cases (%)]	NAT10 expression level [cases(%)]		χ2	*P*
		LOW	High		
**Total**	75 (100)	40 (53.3)	35 (46.7)		
**Gender**				0.008	0.929
Female	44 (58.7)	23 (52.3)	21 (47.7)		
Male	31 (41.3)	17 (53.3)	14 (46.7)		
**Age (years)**				1.804	0.179
<65	31 (41.3)	10 (40)	21 (60)		
>65	44 (58.7)	25 (56.8)	19 (43.2)		
**OS**				0.054	0.816
<12	38 (50.7)	20 (51.4)	18 (48.6)		
>12	37 (49.3)	20 (54.1)	17 (45.9)		
**PFS**				5.825	0.016
<10	43 (57.3)	18 (40.5)	25 (59.5)		
>10	32 (42.7)	22 (68.8)	10 (31.3)		
**T stage**				0.456	0.500
T1-T2	48 (64)	27 (56.3)	21 (43.8)		
T3-T4	27 (36)	13 (48.1)	14 (51.9)		
**Pathological stage**				0.029	0.865
Ⅰ-Ⅱ	69 (92)	37 (53.6)	32 (46.4)		
Ⅲ-Ⅳ	6 (8)	3 (50)	3 (50)		
**Tumor Site**				0.163	0.983
Pancreatic Neck	6 (8)	3 (50)	3 (50)		
Pancreatic Body	12 (16)	7 (58.3)	5 (41.7)		
Pancreatic Head	42 (56)	22 (52.4)	20 (47.6)		
Pancreatic Tail	15 (20)	8 (53.3)	7 (46.7)		
**Differentiation**				1.418	0.492
Poor	20 (26.7)	9 (45)	11 (55)		
Well	11 (14.7)	5 (45.5)	6 (54.5)		
Moderate	44 (58.7)	26 (59.1)	18 (40.9)		
**Perineural Invasion**				0.06	0.807
Present	58 (77.3)	31 (53.4)	27 (46.6)		
Absent	17 (22.7)	9 (52.94)	8 (47.06)		
**Vascular Invasion**				3.446	0.063
Present	20 (26.7)	7 (35)	13 (65)		
Absent	55 (73.3)	33 (59.3)	22 (40.7)		

### NAT10 promotes the malignant progression of pancreatic cancer in vitro

**Fig. 2:**
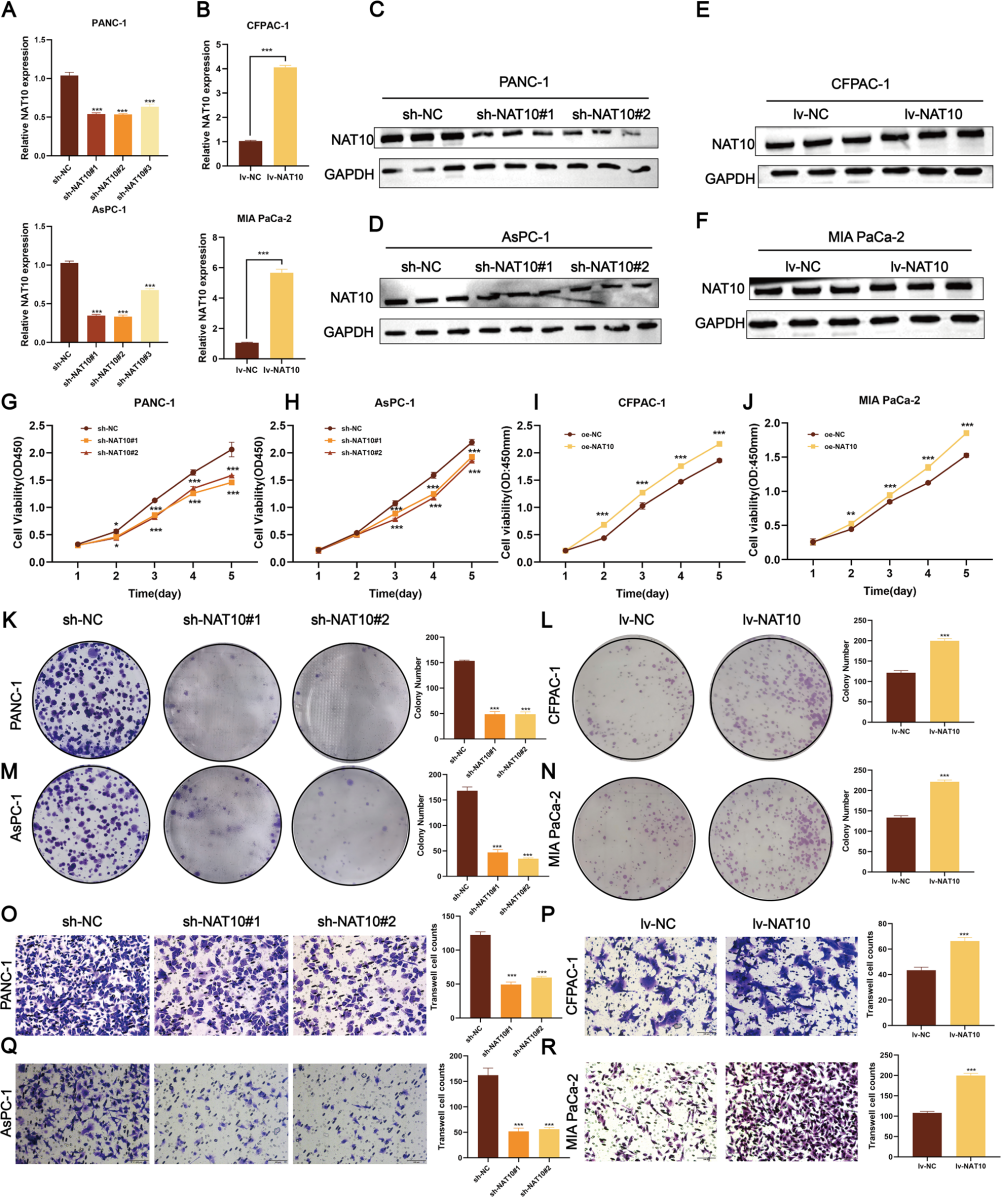
NAT10 promotes pancreatic cancer proliferation and metastasis. **A** Knockdown efficiency of NAT10 mRNA in PANC-1 and AsPC-1 cells. NAT10 mRNA levels were significantly lower in the sh-NAT10#1 and sh-NAT10#2 groups than in the sh-NC group (*P* < 0.001).**B** Efficiency of NAT10 mRNA overexpression in CFPAC-1 and MIA PaCa-2 cells. NAT10 mRNA levels were significantly increased in lv-NAT10 cells compared with lv-NC controls (*P* < 0.001). **C**, **D** Western blot analysis confirmed significant knockdown of NAT10 protein in PANC-1 and AsPC-1 cells. **E**, **F** Western blot analysis revealed significantly elevated NAT10 protein levels in lv-NAT10 CFPAC-1 and MIA PaCa-2 cells compared with lv-NC controls. **G**, **H** NAT10 knockdown significantly inhibited the viability of PANC-1 and AsPC-1 cells over 5 days (*P* < 0.001). **I**, **J** NAT10 overexpression significantly promoted the viability of CFPAC-1 and MIA PaCa-2 cells over 5 days (*P* < 0.001). **K**–**N** Colony formation assays revealed that NAT10 knockdown reduced colony numbers in PANC-1 and AsPC-1 cells (*P* < 0.001), whereas NAT10 overexpression increased colony numbers in CFPAC-1 and MIA PaCa-2 cells (*P* < 0.001). **O**–**R** Transwell assays revealed that NAT10 knockdown significantly reduced the migration of PANC-1 and AsPC-1 cells (*P* < 0.001), whereas NAT10 overexpression enhanced the migratory ability of CFPAC-1 and MIA PaCa-2 cells (*P* < 0.001).

To assess the role of NAT10 in pancreatic cancer, cell lines with high NAT10 expression (PANC-1 and AsPC-1) and low NAT10 expression (CFPAC-1 and MIA PaCa-2) were selected. Lentiviral transduction was used to generate stable NAT10-knockdown and NAT10-overexpressing cell lines. qPCR analysis confirmed that NAT10 knockdown significantly reduced NAT10 mRNA levels in PANC-1 and AsPC-1 cells (Fig. 2A), whereas NAT10 overexpression markedly increased the NAT10 mRNA levels in CFPAC-1 and MIA PaCa-2 cells (Fig. 2B). Western blot analysis further validated the changes in the NAT10 protein levels (Fig. 2C–F). Functional assays demonstrated that NAT10 enhanced cell proliferation, with CCK-8 assays showing reduced cell viability after NAT10 knockdown (Fig. 2G, H) and increased viability following NAT10 overexpression (Fig. 2I, J). Colony formation assays supported these findings, with decreased colony formation after NAT10 knockdown (Fig. 2K, M) and increased colony numbers after NAT10 overexpression (Fig. 2L, N).

Transwell assays revealed that NAT10 knockdown significantly inhibited the migration and invasion of PANC-1 and AsPC-1 cells (Fig. 2O, Q), whereas NAT10 overexpression increased migration and invasion of CFPAC-1 and MIA PaCa-2 cells (Fig. 2P, R). Collectively, these results demonstrated that NAT10 promotes pancreatic cancer cell proliferation, migration, and invasion in vitro, highlighting its critical role in malignant progression.

### NAT10 promotes malignant progression of pancreatic cancer and modulates the immune microenvironment in vivo

**Fig. 3:**
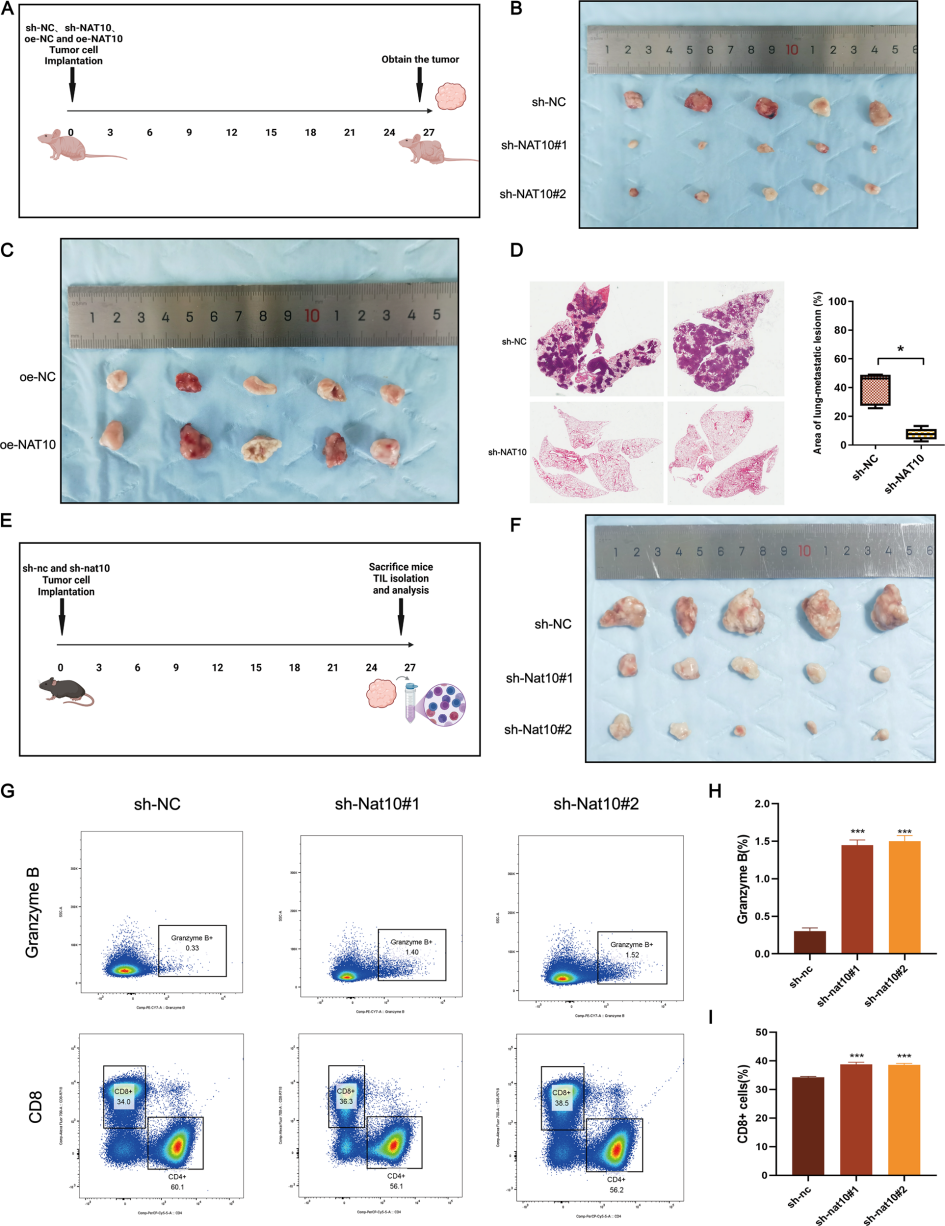
NAT10 promotes the proliferation and metastasis of pancreatic cancer in vivo and inhibits CD8 + T cell infiltration. **A** Schematic diagram of the nude mouse subcutaneous tumor model used to evaluate the effects of NAT10 knockdown or overexpression on pancreatic cancer progression. **B** NAT10 knockdown significantly reduced tumor weight in the nude mouse subcutaneous tumor model (*P* < 0.001). **C** NAT10 overexpression significantly increased tumor weight in the nude mouse subcutaneous tumor model (*P* < 0.001). **D** NAT10 knockdown significantly reduced the lung metastasis area in the nude mouse tail vein lung metastasis model (*P* < 0.05). **E** Schematic diagram of the immunocompetent C57BL/6 mouse subcutaneous tumor model used to assess changes in tumor-infiltrating lymphocytes (TILs) after NAT10 knockdown. **F** NAT10 knockdown significantly reduced tumor weight in the C57BL/6 mouse subcutaneous tumor model (*P* < 0.001). **G**–**I** Flow cytometry analysis revealed that NAT10 knockdown significantly increased CD8 + T-cell infiltration and granzyme B expression in pancreatic cancer tumors (*P* < 0.001).

Using a subcutaneous transplantation tumor model with NAT10-knockdown PANC-1 cells and NAT10-overexpressing MIA PaCa-2 cells (Fig. 3A), we observed that NAT10 knockdown significantly reduced tumor growth and weight (Fig. 3B, S1A), whereas NAT10 overexpression markedly enhanced tumor growth (Fig. 3C, S1C). NAT10 knockdown significantly reduced the number of lung metastases derived from PANC-1 cells in vivo (Fig. 3D). The body weights of all nude mouse model animals were consistent(Fig. S1B, D, E).These findings underscore the critical role of NAT10 in the promotion of pancreatic cancer growth and metastasis.

Similar results were obtained in immunocompetent C57BL/6 J mice (Fig. 3E, F, S1F). The body weight remained consistent across all groups (Fig. S1G).Flow cytometry analysis revealed that NAT10 knockdown significantly increased CD8 + T cell infiltration and granzyme B levels, indicating enhanced anti-tumor immunity (Fig. 3G–I). These findings suggest that NAT10 suppresses immune responses and promotes pancreatic cancer growth.

### NAT10 regulates PD-L1 expression to suppress the immune microenvironment in pancreatic cancer

**Fig. 4:**
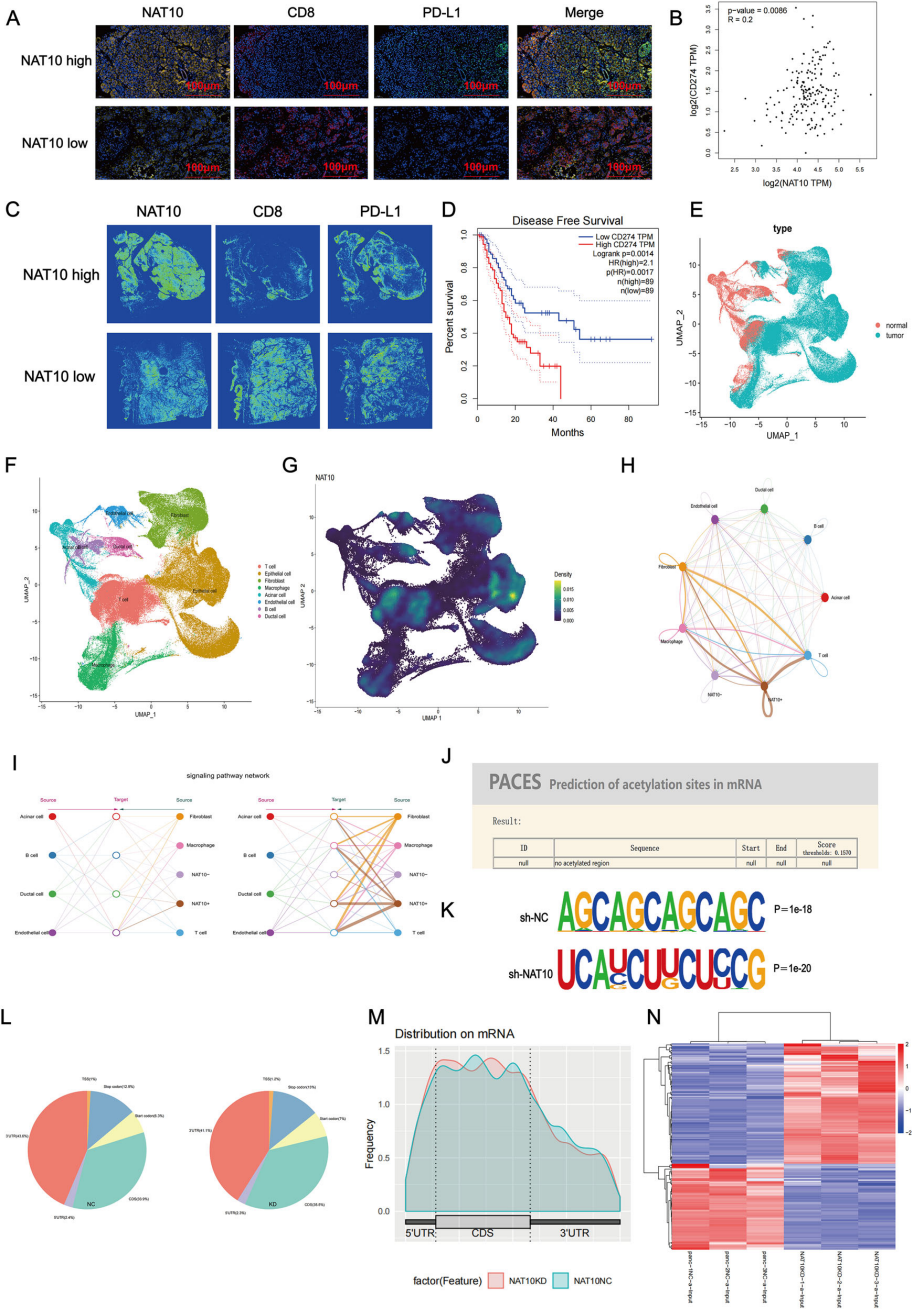
NAT10 indirectly regulates PD-L1 expression through ac4C acetylation. **A** Multiplex immunohistochemistry (mIHC) reveals the expression and spatial distribution of NAT10, CD8, and PD-L1 in pancreatic cancer tissues. **B** Correlation analysis (GEPIA) revealed a positive correlation between NAT10 and PD-L1 expression (*P* = 0.0086, R = 0.2). **C** Heatmap analysis showing the density and colocalization of NAT10, CD8, and PD-L1 in pancreatic cancer tissues. **D** High PD-L1 expression is associated with reduced disease-free survival (DFS) in pancreatic cancer patients (GEPIA database). **E** The UMAP plot illustrates the distribution of tumor and normal cells in the single-cell data. **F** The UMAP plot illustrates the distribution of T cell, Epithelial cell, Fibroblast, Macrophage, Acinar cell, Endothelial cell, B cell, and Ductal cell in the single-cell data. **G** The UMAP plot illustrates the distribution of NAT10 across different cells in the single-cell data. **H** The cell-cell communication plot illustrates the interactions between epithelial cells with high and low expression of NAT10 and different cells. **I** The hierarchical plot illustrates the interactions between epithelial cells with high and low expression of NAT10 and different cells. **J** PACES analysis revealed the absence of ac4C acetylation sites on PD-L1 mRNA. **K** Consensus ac4C motifs identified in control (sh-NC) and NAT10-knockdown (sh-NAT10) cells via HOMER analysis. **L** Pie charts showing the distribution of ac4C peaks across RNA regions (5’UTR, CDS, 3’UTR) in control and NAT10-knockdown cells. **M** Metagene analysis depicts the ac4C peak distribution across RNA regions in control and NAT10-knockdown cells. **N** Heatmap visualization of differentially acetylated transcripts between the control and NAT10-knockdown groups.

Immunohistochemistry (IHC) analysis revealed that NAT10 could inhibit the infiltration of CD8 + T cells in pancreatic cancer tissues, suggesting its immunosuppressive role. Tumor cells often exploit interactions between PD-L1 and PD-1 to evade CD8 + T cell-mediated immune surveillance. Based on these findings, we speculated that NAT10 might regulate PD-L1 to mediate immune evasion in pancreatic cancer. Multiplex IHC staining and density heat maps demonstrated the co-expression of NAT10 and PD-L1, which was inversely related to the expression of CD8 + T cells (Fig. 4A, C). GEPIA revealed a positive correlation between NAT10 and PD-L1 expression (Fig. 4B). Moreover, higher PD-L1 expression was associated with worse prognosis (Fig. 4D), which was consistent with the disease-free survival (DFS) results of NAT10 in pancreatic cancer. qPCR analysis of NAT10 - knocked - down and NAT10 - overexpressing cells further confirmed a positive correlation between NAT10 and PD-L1 mRNA levels (Fig. S2A, B).

To gain further insight into the role of NAT10 in pancreatic cancer, we used single-cell sequencing data. After clustering and annotation, the distribution of cells in reduced-dimensional space was visualized using UMAP plots. The results showed that pancreatic cancer tissues contained various cell types including T cells, B cells, plasma cells, myeloid cells, epithelial cells, fibroblasts, and endothelial cells (Fig. 4E–G). Notably, NAT10 was significantly expressed in epithelial cells, T cells, and fibroblasts, with the highest expression observed in tumor epithelial cells. This finding further suggests that NAT10 may play a crucial role in tumor epithelial cells, promoting the generation of an immunosuppressive microenvironment in pancreatic cancer, which is in line with our research direction. Cell–cell communication analysis revealed that compared to tumor epithelial cells with low NAT10 expression, those with high NAT10 expression had more intense interactions with T cells, macrophages, and fibroblasts, especially with T cells (Fig. 4H, I). The results of the cell-cell communication analysis indicated that NAT10 might inhibit the pancreatic cancer immune microenvironment by affecting T cell function, which was consistent with the results of our pathological staining and further suggested that NAT10 might inhibit the infiltration of CD8 + T cells through PD-L1.

### NAT10 indirectly regulates PD-L1-mediated immune evasion in pancreatic cancer

Our previous results, obtained using mIHC and qPCR, demonstrated a positive correlation between NAT10 and PD-L1. As PD-L1 serves as a key immune checkpoint in tumors, we explored whether NAT10 regulates PD-L1 expression via ac4C. Interestingly, PACES [26] analysis revealed no direct ac4C acetylation sites in PD-L1 mRNA (Fig. 4J). To further investigate the potential ac4C acetylation of PD-L1 mRNA in pancreatic cancer cells, RNA-seq and acRIP-seq analyses were conducted. The acRIP-seq results showed significant enrichment of ac4C motifs in the gene sequences (Fig. 4K). Differential expression analysis following NAT10 knockdown revealed extensive transcriptomic changes (Fig. 3H–J). However, PD-L1 mRNA does not contain ac4C acetylation sites. Therefore, we focused on whether NAT10 indirectly regulates PD-L1 expression through ac4C acetylation or other mechanisms to inhibit the infiltration of CD8 + T cells.

### KRT8 promotes pancreatic cancer proliferation in vitro

Although no ac4C acetylation sites were found on PD-L1 using RNA-seq and acRIP-seq, we discovered ac4C acetylation sites on KRT8, a key oncogene, using RNA-seq and acRI-seq. NAT10 may regulate the expression of adhesion molecules on the surface of tumor cells through KRT8, thereby leading to PD-L1-mediated immune evasion in pancreatic cancer. To this end, we investigated whether KRT8 could affect malignant progression and the immune microenvironment of pancreatic cancer by examining its impact on aggressive progression and the immune microenvironment of pancreatic cancer.

**Fig. 5:**
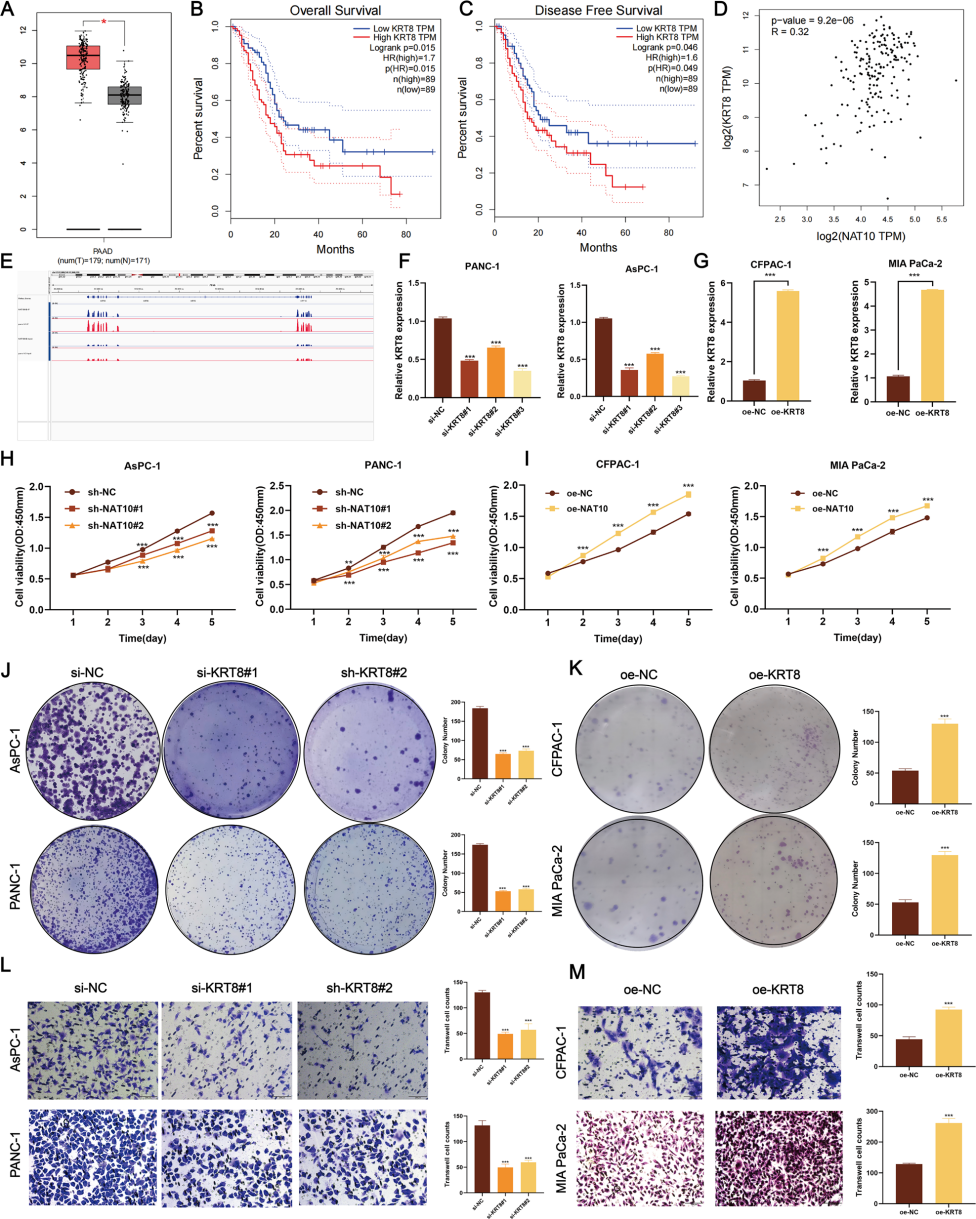
KRT8 promotes the malignant progression of pancreatic cancer. **A** GEPIA revealed significantly elevated KRT8 expression in pancreatic cancer tissues compared with normal tissues (*P* < 0.001). **B**, **C** High KRT8 expression is associated with poor overall survival (OS) and disease-free survival (DFS) in pancreatic cancer patients (GEPIA database). **D** GEPIA revealed a positive correlation between NAT10 and KRT8 expression (*P* = 9.2e-06, R = 0.32). **E** Distribution of ac4C acetylation sites on KRT8 mRNA, as revealed by acRIP-seq. **F**, **G** Validation of KRT8 knockdown and overexpression efficiency at the RNA level in pancreatic cancer cells. KRT8 expression is significantly reduced in si-KRT8#1 and si-KRT8#2 cells and significantly increased in oe-KRT8 cells (*P* < 0.001). **H**, **I** KRT8 knockdown significantly inhibits the proliferation of AsPC-1 and PANC-1 cells (*P* < 0.001), whereas KRT8 overexpression promotes the proliferation of CFPAC-1 and MIA PaCa-2 cells (*P* < 0.001). **J**, **K** Colony formation assays demonstrate that KRT8 knockdown reduces the number of colonies formed by AsPC-1 and PANC-1 cells (*P* < 0.001), whereas KRT8 overexpression increases colony formation in CFPAC-1 and MIA PaCa-2 cells (*P* < 0.001). **L**, **M** Transwell assays show that KRT8 knockdown inhibits the migration of AsPC-1 and PANC-1 cells (*P* < 0.001), whereas KRT8 overexpression enhances the migratory capacity of CFPAC-1 and MIA PaCa-2 cells (*P* < 0.001).

GEPIA revealed that KRT8 is highly expressed in pancreatic cancer tissues compared with normal tissues (Fig. 5A) and is significantly associated with poor overall and disease-free survival (Fig. 5B, C). Moreover, NAT10 expression positively correlated with KRT8 expression (Fig. 5D), suggesting a functional relationship.

Combined RNA-seq and acRIP-seq analyses revealed ac4C acetylation sites in KRT8 mRNA (Fig. 5E), indicating that NAT10-mediated ac4C modification regulates KRT8 expression. qPCR analysis confirmed efficient knockdown and overexpression of KRT8 in pancreatic cancer cells (Fig. 5F, G). Functional assays demonstrated that KRT8 promoted pancreatic cancer cell proliferation, and CCK-8 assays revealed reduced viability upon KRT8 knockdown (Fig. 5H), and increased viability upon KRT8 overexpression (Fig. 5I). The results of the colony formation assays corroborated these results, as the number of colonies decreased with KRT8 knockdown (Fig. 5J), but increased with KRT8 overexpression (Fig. 5K).

### KRT8 promotes pancreatic cancer metastasis in vitro

Transwell assays revealed that KRT8 knockdown significantly impaired cell migration and invasion (Fig. 5L), whereas KRT8 overexpression enhanced these abilities (Fig. 5M). Collectively, these findings highlight the critical role of KRT8 in promoting the malignant progression of pancreatic cancer. NAT10-mediated ac4C acetylation of KRT8 mRNA stabilizes its expression, contributing to tumor growth, migration, and invasion.

### NAT10 promotes pancreatic cancer progression by regulating KRT8 through ac4C acetylation

**Fig. 6:**
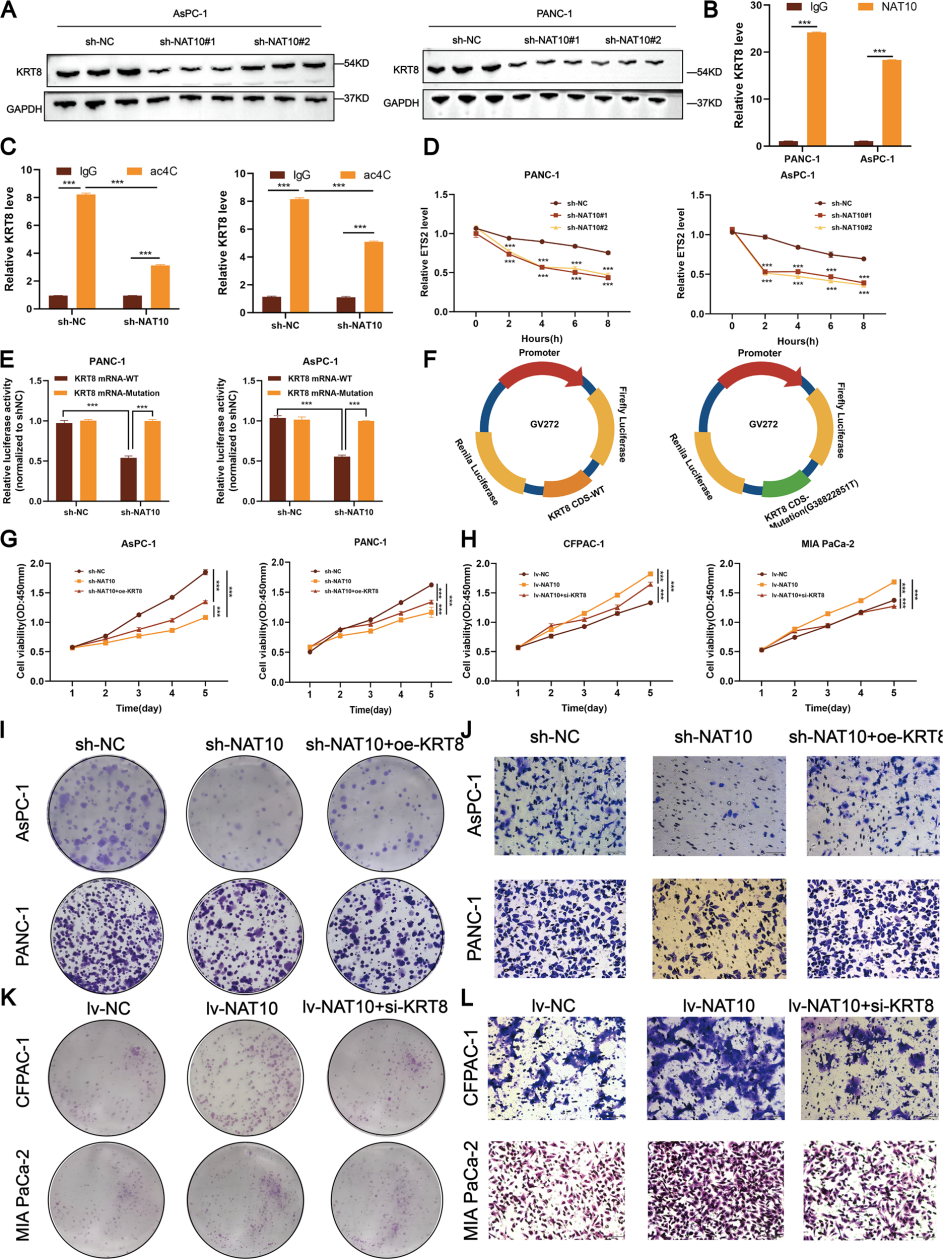
NAT10 regulates KRT8 through ac4C acetylation to promote pancreatic cancer proliferation and metastasis. **A** Western blot analysis revealed reduced KRT8 protein levels in AsPC-1 and PANC-1 cells after NAT10 knockdown. **B** RIP-qPCR revealed that NAT10 directly binds to KRT8 mRNA in PANC-1 and AsPC-1 cells, with significantly reduced binding in NAT10-knockdown cells (*P* < 0.001). **C** acRIP-qPCR revealed lower ac4C acetylation levels on KRT8 mRNA in NAT10-knockdown cells than in control cells (*P* < 0.001). **D** Actinomycin D chase assays revealed decreased KRT8 mRNA stability in NAT10-knockdown cells (*P* < 0.001). **E** Dual-luciferase reporter assays revealed that NAT10 knockdown significantly reduced luciferase activity in constructs containing wild-type KRT8 mRNA sequences but not in constructs with ac4C site mutations (*P* < 0.001). **F** Schematic representation of the wild-type and mutant KRT8 mRNA constructs used for the luciferase reporter assays. **G**, **H** NAT10 knockdown inhibits pancreatic cancer cell proliferation, whereas KRT8 overexpression rescues this effect (*P* < 0.001). **I** Colony formation assays revealed that NAT10 knockdown reduces colony formation in AsPC-1 and PANC-1 cells and that KRT8 overexpression rescues this effect (*P* < 0.001). **J** Transwell assays revealed that NAT10 knockdown inhibits the migration of AsPC-1 and PANC-1 cells, whereas KRT8 overexpression restores migratory capacity (*P* < 0.001). **K** The colony formation assay demonstrated that NAT10 overexpression increased colony formation in MIA PaCa-2 and CFPAC-1 cells, while KRT8 knockdown rescued this effect (*P* < 0.001). **L** The Transwell assay revealed that NAT10 overexpression enhanced the migration of MIA PaCa-2 and CFPAC-1 cells, whereas KRT8 knockdown suppressed the migratory capacity (*P* < 0.001).

Western blotting(Fig. 6A). and qPCR (Fig. S2D–F) analyses revealed significantly reduced KRT8 protein and mRNA levels after NAT10 knockdown. RIP-qPCR experiments confirmed that NAT10 binds directly to KRT8 mRNA (Fig. 6B), and acRIP-qPCR demonstrated the specific ac4C acetylation of KRT8 mRNA (Fig. 6C). RNA stability assays revealed that KRT8 mRNA stability was significantly reduced in NAT10-knockdown cells (Fig. 6D).

Dual-luciferase reporter assays confirmed that NAT10 knockdown reduced luciferase activity in the wild-type KRT8 construct but not in the mutated construct lacking the ac4C site (Fig. 6E, F). Functional assays revealed that KRT8 knockdown inhibited pancreatic cancer cell proliferation and migration, whereas NAT10 knockdown and KRT8 overexpression restored these phenotypes, and overexpression of NAT10 combined with KRT8 knockdown also restored these phenotypes (Fig. 6G–L, S3A–H).These results indicate that NAT10-mediated ac4C acetylation enhances KRT8 mRNA stability and expression, thereby promoting pancreatic cancer progression.

### ETS2 mediates the regulatory effects of NAT10 on PD-L1

**Fig. 7:**
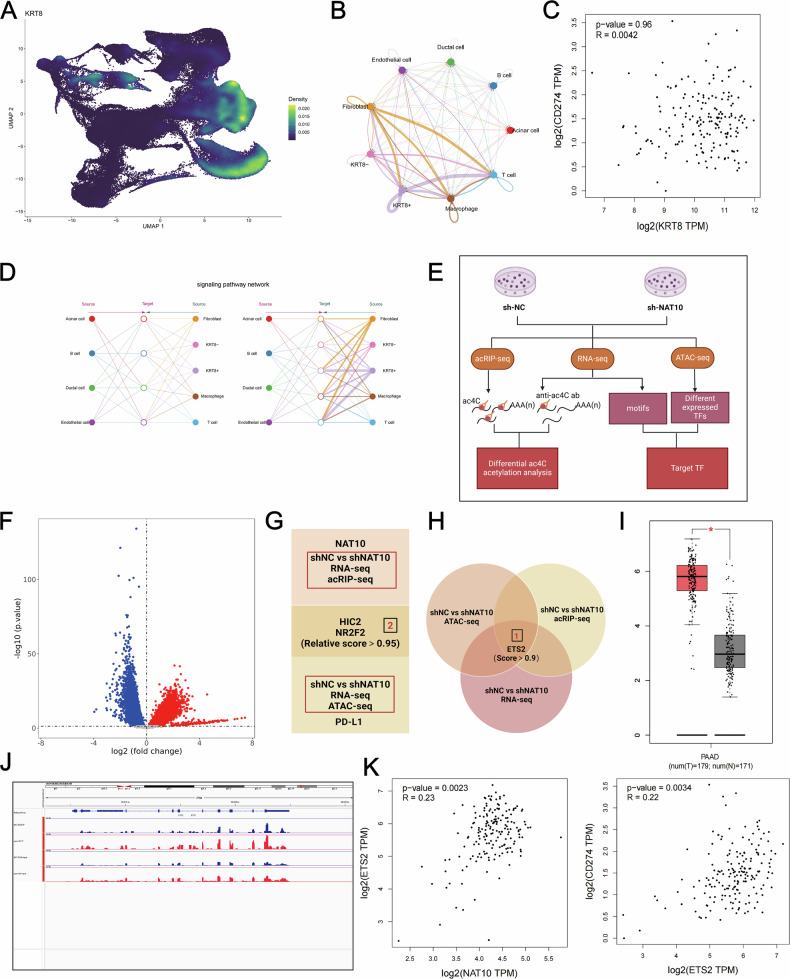
ETS2 is the key gene through which NAT10 indirectly regulates PD-L1 via ac4C acetylation. **A** The UMAP plot illustrates the distribution of KRT8 across different cells in the single-cell data. **B** The cell-cell communication plot illustrates the interactions between epithelial cells with high and low expression of KRT8 and different cells. **C** Correlation analysis (GEPIA) revealedassociations between KRT8 and CD274 (*P* = 0.96, R = 0.0042). **D** The hierarchical plot illustrates the interactions between epithelial cells with high and low expression of KRT8 and different cells. **E** Workflows of RNA-seq, acRIP-seq, and ATAC-seq showing the identification of target transcription factors (TFs) regulated by NAT10. **F** Volcano plot showing chromatin accessibility changes in NAT10-knockdown cells (ATAC-seq analysis). **G** Conjoint analysis identified key transcription factors, including ETS2, through RNA-seq, acRIP-seq, and ATAC-seq integration. **H** Venn diagram showing the overlap of differentially expressed transcription factors and acetylated transcripts, highlighting ETS2 as a central target. **I** GEPIA revealed significantly elevated ETS2 expression in pancreatic cancer tissues compared with normal tissues. **J** acRIP-seq peak visualization of ETS2 mRNA showing ac4C enrichment in control cells. **K** Correlation analysis (GEPIA) revealed positive associations between ETS2 and NAT10 (*P* = 0.0023, R = 0.22) as well as between ETS2 and PD-L1 (*P* = 0.0034, R = 0.22).

Single-cell analysis of KRT8 revealed that KRT8 was predominantly highly expressed in tumor epithelial cells (Fig. 7A). Furthermore, compared to tumor epithelial cells with low KRT8 expression, those with high KRT8 expression exhibited stronger interactions with T cells (Fig.7B, D). Interestingly, these results were consistent with findings related to NAT10. However, no correlation was observed between KRT8 and PD - L1 expression (Fig. 7C). This indicates that, although NAT10 can enhance the interaction with T cells through ac4C acetylation of KRT8, it cannot influence PD -L1 - mediated immune suppression via KRT8.

We performed RNA-seq and acRIP-seq to identify the downstream targets regulated by NAT10, and used ATAC-seq to explore the upstream regulators of PD-L1 (Fig. 7E). ATAC-seq analysis revealed changes in chromatin accessibility between NAT10-knockdown cells and control cells (Fig. 7F). However, HIC and NR2F2 levels did not show a positive correlation with NAT10 and PD-L1 (Fig. 7G). To address this, we expanded the number of genes examined. Interestingly, by integrating the results of the three sequencing methods, we found that ETS2 contains ac4C sites (Fig. 7J) and can bind to the promoter regions of NAT10 and PD-L1 (Fig. 7H), forming a regulatory loop that further enhances NAT10’s regulation of PD by L1. GEPIA confirmed that ETS2 was highly expressed in pancreatic cancer (Fig. 7I) and positively correlated with the expression of NAT10 and PD-L1 (Fig. 7K). These results suggested that NAT10 may promote immune suppression in pancreatic cancer by regulating the ETS2–PD-L1 axis through ac4C acetylation.

### NAT10 promotes immune suppression in pancreatic cancer by facilitating the binding of T-cell ligand-receptor through KRT8

We employed single - cell analysis technology to thoroughly examine the binding of NAT10 and KRT8 to T - cell receptors, thereby gaining novel insights into the mechanism underlying NAT10 - regulated KRT8 - mediated immune suppression in pancreatic cancer through ac4C acetylation. Our single-cell ligand-receptor bubble plot analysis showed that in pancreatic cancer epithelial cells, the expression levels of MIF, MDK, and LGALS9 were significantly higher when NAT10 and KRT8 were highly expressed than when they were low-expressed (Fig. S4A, B). To further determine whether NAT10 and KRT8 interact with CD8 + T cells, we performed an in-depth analysis of T-cell subpopulations (Fig. S4C). The subsequent cell-cell communication analysis indicated that, compared with epithelial cells with low expression of NAT10 and KRT8, those with high expression exhibited a significantly enhanced interaction with CD8 + T cells (Fig. S4D, E). Further single-cell ligand-receptor bubble plot analysis suggested that in pancreatic cancer epithelial cells, high expression of NAT10 and KRT8, compared with low expression, not only up - upregulated MIF, MDK, and LGALS9 but also strengthened the interaction with CD8 + T cells, thereby mediating immune evasion(Fig. S4F, G). These results imply that NAT10 may promote the increase of KRT8 ligands in tumor epithelial cells through ac4C acetylation and bind to the corresponding receptors on CD8 + T cells, consequently enhancing the interaction between tumor cells and CD8 + T cells and ultimately suppressing the immune microenvironment. However, the specific molecular mechanisms involved in this process and the cell types concerned still require further in-depth research to be clarified.

### NAT10 regulates ETS2 mRNA stability through ac4C acetylation

**Fig. 8:**
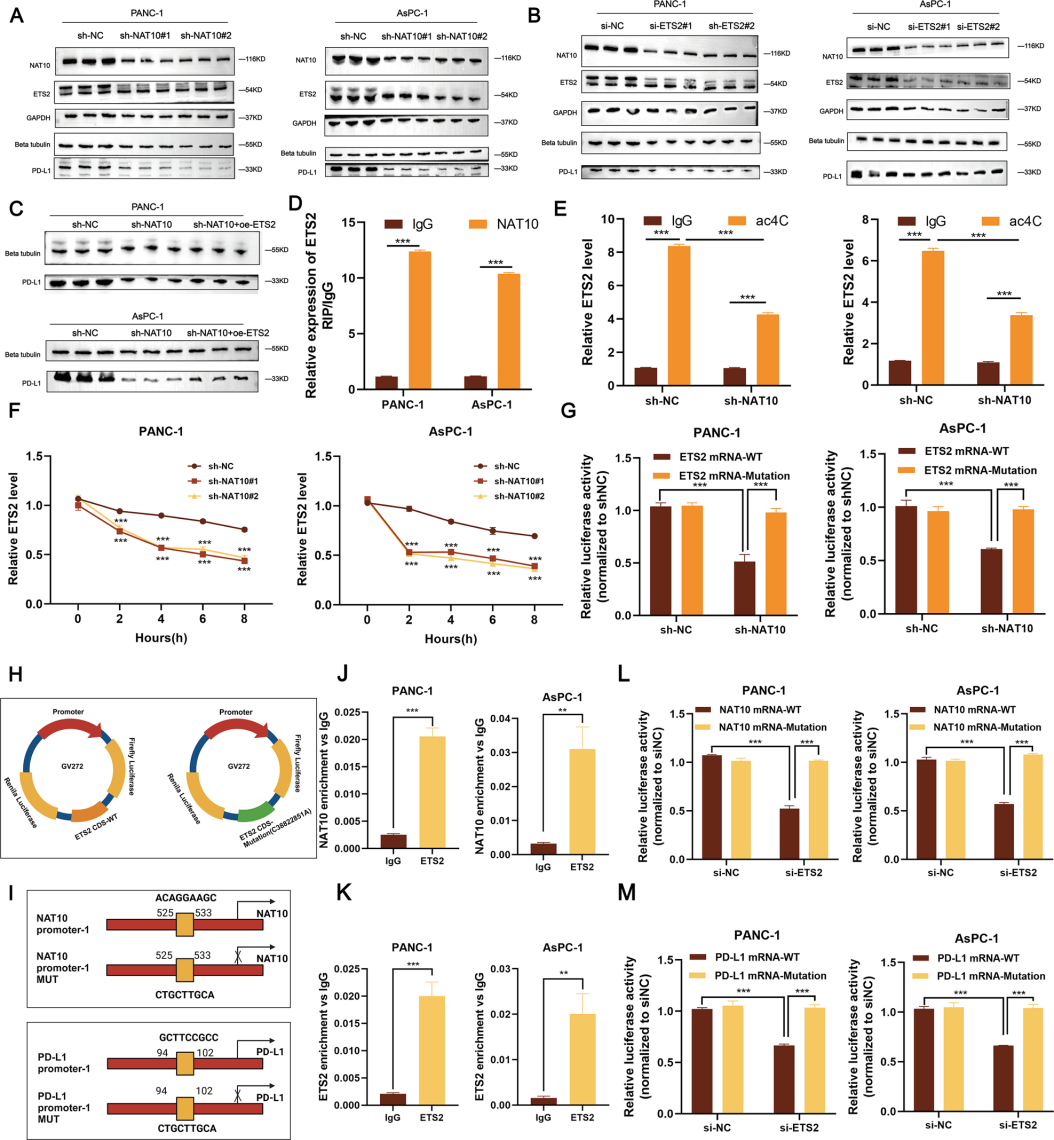
NAT10 regulates PD-L1 through ac4C acetylation of ETS2, and ETS2 promotes NAT10 transcription. **A** NAT10 knockdown reduces ETS2 and PD-L1 protein levels in PANC-1 and AsPC-1 cells, as shown by Western blot analysis. **B** ETS2 knockdown decreases NAT10 and PD-L1 protein levels in PANC-1 and AsPC-1 cells, indicating a reciprocal regulatory relationship between NAT10 and ETS2. **C** ETS2 overexpression restores PD-L1 protein expression in NAT10-knockdown cells, confirming that NAT10 regulates PD-L1 via ETS2. **D** RIP-qPCR revealed that NAT10 directly binds to ETS2 mRNA, with significantly reduced binding in NAT10-knockdown cells (*P* < 0.001). **E** acRIP-qPCR revealed reduced ac4C acetylation levels on ETS2 mRNA in NAT10-knockdown cells (*P* < 0.001). **F** Actinomycin D chase assays demonstrated reduced ETS2 mRNA stability in NAT10-knockdown cells compared with control cells (*P* < 0.001). **G** Dual-luciferase reporter assays revealed that NAT10 knockdown significantly reduced luciferase activity in constructs with wild-type ETS2 mRNA sequences but not in those with ac4C site mutations (*P* < 0.001). **H** Schematic representation of ETS2 mRNA constructs used in dual-luciferase reporter assays, including wild-type and ac4C site-mutated sequences. **I** Schematic representation of wild-type and mutant ETS2-binding sequences in the NAT10 and PD-L1 promoters used for luciferase reporter assays. **J** ChIP‒qPCR revealed significant enrichment of ETS2 binding to the NAT10 promoter in PANC-1 and AsPC-1 cells (*P* < 0.001). **K** ChIP‒qPCR revealed that ETS2 binds directly to the PD-L1 promoter in PANC-1 and AsPC-1 cells (*P* < 0.001). **L** Dual-luciferase reporter assays revealed that ETS2 knockdown significantly reduced luciferase activity in the constructs containing the wild-type NAT10 promoter but not in the mutated constructs (*P* < 0.001). **M** Luciferase activity was significantly reduced in constructs containing the wild-type PD-L1 promoter after ETS2 knockdown, with no effect observed in the mutant constructs (*P* < 0.001).

Western blot analysis revealed that NAT10 knockdown significantly reduced ETS2 and PD-L1 protein levels in PANC-1 and AsPC-1 cells (Fig. 8A), indicating that NAT10 positively regulated both proteins. Similarly, ETS2 knockdown decreased the NAT10 and PD-L1 levels (Fig. 8B), suggesting a reciprocal regulatory relationship between NAT10 and ETS2. Overexpression of ETS2 in NAT10-knockdown cells partially restored PD-L1 levels (Fig. 8C), further confirming that NAT10 regulates PD-L1 expression via ETS2. These findings are consistent with the PCR results (Fig. S4A–H).

RNA immunoprecipitation (RIP‒qPCR) demonstrated that NAT10 directly binds to ETS2 mRNA (Fig. 8D), and ac4C‒qPCR analysis revealed that NAT10 knockdown significantly reduced ac4C acetylation of ETS2 mRNA (Fig. 8E). RNA stability assays revealed that ETS2 mRNA stability decreased over time in NAT10-knockdown cells (Fig. 8F). Dual-luciferase reporter assays confirmed that NAT10 knockdown significantly reduced luciferase activity in the wild-type ETS2 construct but not in the mutated ac4C construct (Fig. 8G–H). These results demonstrate that NAT10 stabilizes ETS2 mRNA by catalyzing ac4C acetylation, thereby regulating PD-L1 expression and contributing to the immunosuppressive microenvironment of pancreatic cancer.

### ETS2 promotes the transcription of NAT10 and PD-L1

Western blotting analysis revealed that ETS2 knockdown reduced the protein levels of both NAT10 and PD-L1 in pancreatic cancer cells (Fig. 8B). ChIP‒qPCR experiments confirmed that ETS2 binds directly to the promoter regions of NAT10 and PD-L1, thereby promoting their transcription (Fig. 8I–K). Dual-luciferase reporter assays using wild-type or mutated promoter constructs of NAT10 and PD-L1 revealed that ETS2 knockdown significantly reduced luciferase activity in wild-type constructs, whereas the mutant constructs were unaffected (Fig. 8L, M). Recovery experiments demonstrated that ETS2 overexpression rescued PD-L1 expression in NAT10-knockdown cells (Fig. 8C). These findings indicate that ETS2 acts as a transcriptional activator of both NAT10 and PD-L1, forming a positive feedback loop that amplifies immune suppression and promotes pancreatic cancer progression.

### Targeting NAT10 combined with PD-L1 blockade inhibits pancreatic cancer progression

**Fig. 9:**
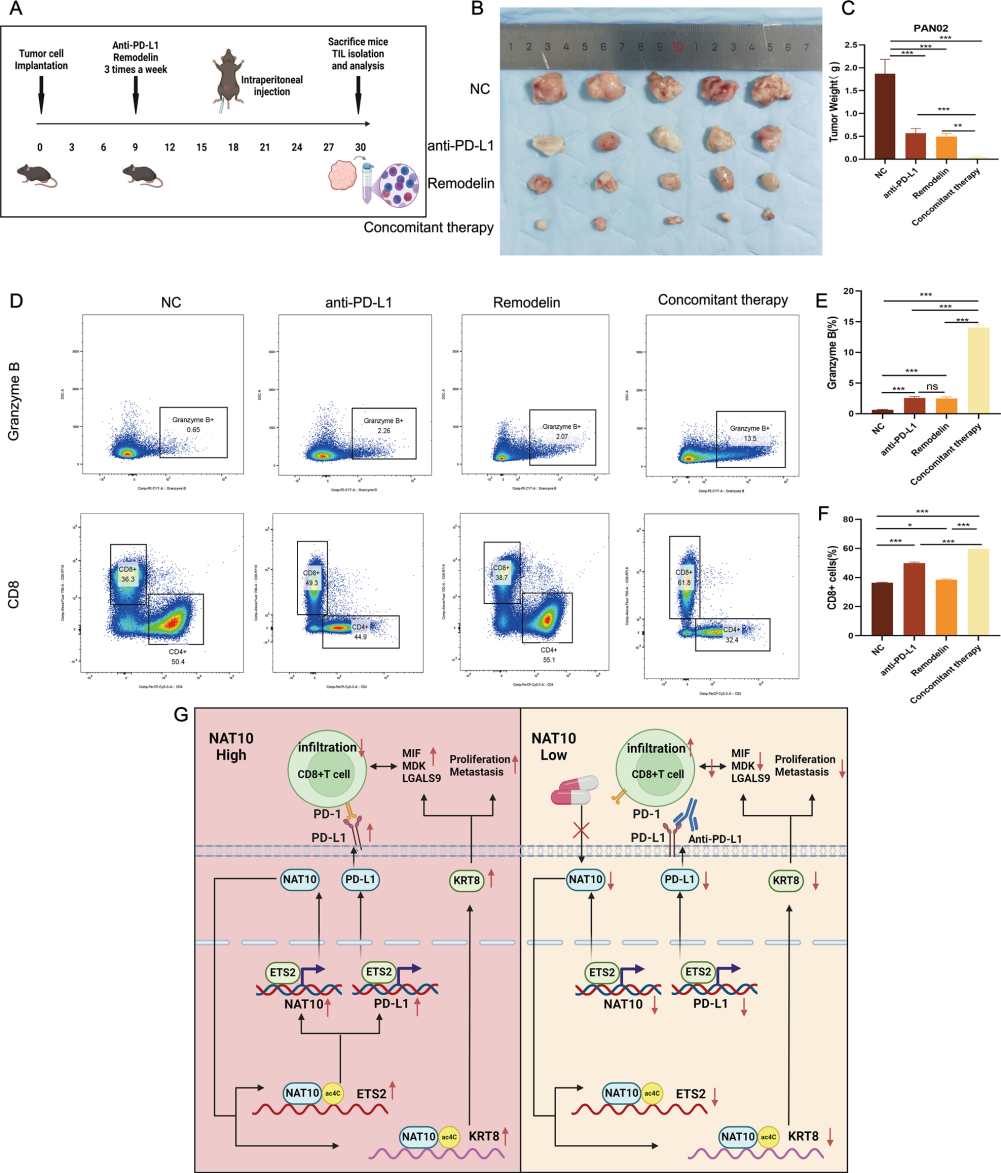
Targeting NAT10 combined with anti-PD-L1 therapy improves the immune microenvironment and inhibits pancreatic cancer progression. **A** Schematic diagram of the combination therapy mouse model using an anti-PD-L1 antibody and Remodelin. **B**, **C** Compared with single agent therapy, combination therapy significantly reduced tumor weight (*P* < 0.001). **D**–**F** Flow cytometry analysis demonstrated that, compared with monotherapy, combination therapy significantly increased CD8 + T-cell infiltration and granzyme B expression (*P* < 0.001). **G** Graphical abstract depicting how NAT10 promotes pancreatic cancer immunosuppression and malignant progression through ac4C acetylation-mediated regulation of ETS2 and KRT8, highlighting its therapeutic potential in combination with anti-PD-L1 therapy.

We hypothesized that inhibition of NAT10 could increase the efficacy of PD-L1/PD-1 blockade immunotherapy. Compared with monotherapy or control, combination therapy with an NAT10 inhibitor and an anti-PD-L1 antibody significantly inhibited tumor growth (Fig. 9A–C). Body weight remained stable across all groups (Fig. S4I). Flow cytometry analysis demonstrated that combination therapy significantly increased CD8 + T-cell infiltration and granzyme B levels (Fig. 9D–F), indicating enhanced antitumor immunity. These results highlight the potential of combining NAT10 inhibition with PD-L1 blockade to improve the therapeutic outcomes in patients with pancreatic cancer.

## Discussion

In this study, we identified NAT10 as a key regulator of pancreatic cancer (PC) progression and immune evasion, primarily through ac4C acetylation-mediated RNA modification. Specifically, NAT10 enhances the stability of ETS2 and KRT8 mRNA via ac4C modification, thereby promoting tumor malignancy and immunosuppression. Further analysis revealed that NAT10 inhibition, in combination with PD-L1 blockade, exhibited a synergistic antitumor effect, offering a promising therapeutic strategy to overcome the immunosuppressive tumor microenvironment (TME) in PC(Fig. 9G).

NAT10 is significantly overexpressed in pancreatic cancer tissues and is closely associated with poor prognosis, which is consistent with its oncogenic role in other malignancies, such as cervical [20] and liver cancers [27]. Mechanistically, NAT10 catalyzes ac4C acetylation of ETS2 mRNA, enhancing its stability and establishing a positive feedback loop that further amplifies NAT10 and PD-L1 expression. This cascade suppresses CD8 + T cell infiltration, ultimately facilitating immune evasion. The NAT10-ETS2-PD-L1 axis not only reveals a novel molecular mechanism linking RNA modification to immune evasion but also underscores NAT10’s central role in shaping the immunosuppressive TME in PC. Notably, NAT10 may influence PD-L1 expression and the TME through non-ac4C-mediated mechanisms. For instance [28], NAT10 could engage in protein-protein interactions with PD-L1 or other immune regulatory proteins, altering their stability or subcellular localization independent of RNA modification. Additionally, NAT10 might modulate post-translational modifications of PD-L1 via indirect signaling cascades, further fine-tuning its expression or functional activity in the TME.

ETS2 (ETS proto-oncogene 2), located on human chromosome 21q22.2, encodes a transcription factor involved in cell proliferation, differentiation, apoptosis, and development [29, 30]. ETS2 regulates gene transcription by binding to specific DNA sequences, thereby influencing cellular behavior and fate [31]. Aberrant ETS2 expression has been observed in various cancers and its function differs across tumor types. In certain malignancies, high ETS2 expression is correlated with increased tumor aggressiveness and poor prognosis [32]. In PC, ETS2 expression is closely linked to tumor progression [33], potentially influencing cell proliferation and apoptosis through the regulation of cell cycle-related genes [34, 35]. It may also play a role in acinar-to-ductal metaplasia (ADM). Additionally, the loss of ETS2 in fibroblasts significantly alters the distribution of immune cells within the TME, characterized by increased CD8 + T-cell infiltration and a reduction in regulatory T cells (Tregs), myeloid-derived suppressor cells (MDSCs), and mature macrophages [36]. In this study, we revealed the role of ETS2 as a transcriptional activator of NAT10 and PD-L1; how ETS2 modulates the immune microenvironment is worthy of in-depth investigations.

KRT8 (keratin 8) is a key intermediate filament protein that is predominantly expressed in simple epithelial tissues and contributes to cellular structural integrity and signaling regulation [29]. Beyond maintaining epithelial mechanical stability, KRT8 is involved in modulating cellular stress responses, apoptosis, and signaling pathways [37]. Aberrant KRT8 expression has been linked to enhanced tumor plasticity, epithelial-mesenchymal transition (EMT), and increased metastatic potential [38]. Previous studies have shown that NAT10 can promote the proliferation, migration, and metastasis of prostate cancer cells by regulating KRT8 through ac4C acetylation [39–41]. We also observed this phenomenon in the pancreas and demonstrated that NAT10 stabilized KRT8 mRNA through ac4C acetylation, thereby driving PC cell proliferation, migration, and invasion, further highlighting the multifaceted regulatory role of NAT10 in PC. Notably, KRT8 is not only highly expressed in pancreatic cancer epithelial cells but also in certain macrophages and fibroblasts, suggesting its potential involvement in mediating intercellular interactions and signaling within the TME.

Single-cell RNA sequencing analysis demonstrated enhanced interactions between pancreatic cancer epithelial cells with high NAT10 and KRT8 expression and T cells. However, whether NAT10-mediated ac4C modification further upregulates KRT8 expression to facilitate epithelial cell ligand T cell receptor binding, suppresses CD8 + T cell infiltration, and ultimately exacerbates immune evasion remains to be elucidated. Additionally, we found that pancreatic cancer epithelial cells with high NAT10 expression have increased interactions with Th1 T cells. Therefore, does NAT10 alter the pancreatic cancer immune microenvironment by affecting Th1 T cells? And what is the specific mechanism? These questions require further investigation. Moreover, the high expression of KRT8 in macrophages and fibroblasts raises an important question: Does NAT10 selectively regulate specific KRT8+ cell subpopulations, forming a physical barrier that impedes immune cell or immunotherapeutic drug penetration? This finding warrants further research.

The intrinsic immune tolerance of PC presents a major challenge for current immunotherapies [42, 43]. Compared to monotherapy, NAT10 inhibition combined with PD-L1 blockade demonstrates superior antitumor efficacy by remodeling the TME [44, 45], enhancing CD8 + T-cell infiltration, and increasing granzyme B levels. This dual-targeting strategy effectively disrupts the immunosuppressive barrier of PC by simultaneously inhibiting the intrinsic tumor driver (NAT10) and extrinsic immune checkpoint (PD-L1). Targeting the NAT10-ETS2-PD-L1 axis while reducing KRT8-mediated tumor plasticity offers a novel approach for overcoming immune resistance and improving patient outcomes.

Despite these significant findings, this study had several limitations. First, it was primarily based on in vitro and animal models, necessitating validation in larger patient cohorts and clinical samples. Second, NAT10 inhibitors (e.g., Remodelin) have shown promising antitumor potential in preliminary experiments. In vitro, Remodelin treatment could suppress the growth of cancer cells but not induce apoptosis, indicating its little cytotoxicity [46]. However, its pharmacological profiles require rigorous evaluation. Preclinical studies indicate that Remodelin may exhibit off-target effects, such as cross-reactivity with other RNA acetyltransferases [47], and dose-limiting toxicities in normal tissues with high NAT10 expression [48]. Additionally, its pharmacokinetic properties, including short half-life and limited tissue penetration in solid tumors, may hinder therapeutic efficacy. These limitations highlight the need for optimizing specificity and safety in future development. Third, the broader RNA network regulated by NAT10 in the TME remains incompletely understood, as it may also influence other immune-related genes or signaling pathways that modulate tumor immunity. Finally, the effects of NAT10 on specific immune cell subsets within the TME, such as Tregs, tumor-associated macrophages, and MDSCs, remain unexplored and warrant further investigation [49, 50].

Future studies should leverage single-cell RNA sequencing to determine whether NAT10 preferentially regulates specific immune and stromal cell subpopulations, and further elucidate its functional mechanisms within the TME. Additionally, exploring the interplay between NAT10 and other epigenetic modifications, such as histone modifications [51] and m6A methylation [52, 53], will provide a more comprehensive understanding of ac4C acetylation in cancer biology. Investigating whether NAT10 promotes immunosuppression by modulating key cytokine signaling pathways [54–56] (e.g., IL-6/STAT3 or TGF-β) in the PC TME will also offer new mechanistic insights. From a translational perspective, advancing NAT10-targeted therapies requires addressing the limitations of current inhibitors: optimizing Remodelin’s structure to enhance specificity for NAT10, improving pharmacokinetics, and developing tumor-selective delivery systems to minimize systemic toxicity. Patient-derived xenograft (PDX) models and pancreatic organoids provide robust preclinical platforms to facilitate clinical translation of these therapeutic strategies.

In conclusion, this study established NAT10 as a central regulator of PC progression and immune evasion through the ac4C-dependent stabilization of ETS2 and KRT8 mRNA, thereby promoting tumor immunosuppression and malignancy. By elucidating the link between RNA modification and immune evasion, our study provides novel insights into the biology of PC and highlights the therapeutic potential of targeting NAT10. The combined inhibition of NAT10 and PD-L1 represents a promising strategy for reshaping the immunosuppressive TME and enhancing antitumor immune responses. Future research should further investigate the role of NAT10 in the PC TME and explore its broader implications in cancer progression and immunotherapy.

## Supplementary information

supplementary table

supplementary figure

qPCR supplementary file

WB supplementary file

## Data Availability

The data will be made available upon request.
