# Supplementary material for: Targeting the ac4C ‘Writer’ NAT10 enhances pancreatic cancer immunotherapy via dual modulation of CD8+ T cells and tumor cells
Source: Cell Death Dis. 2025 Nov 7;16(1):809. doi: 10.1038/s41419-025-08156-0 (PMC12594761; doi:10.1038/s41419-025-08156-0)
Supplement: Supplementary file 1 — supplementary table [file 41419_2025_8156_MOESM1_ESM.docx]

**Table S1 The primer sequences for qPCR**

| Primer |  | SequenCe 5’-3’ |
| --- | --- | --- |
| GAPDH | Forward | GAAGGTGAAGGTCGGAGTC |
|  | Reverse | GAAGATGGTGATGGGATTTC |
| β-actin | Forward | CTCCATCCTGGCCTCGCTGT |
|  | Reverse | GCTGTCACCTTCACCGTTCC |
| NAT10 | Forward | CCACGACGACAGCCAGATTG |
|  | Reverse | CTGAGACTATCCGAGTGATGTTGAG |
| PD-L1 | Forward | TGACCTACTGGCATTTGCTGAACG |
|  | Reverse | CACTGCTTGTCCAGATGACTTCGG |
| ETS2 | Forward | GCTGGACTGGAGACGGATGG |
|  | Reverse | AGTTCTGGAGGTCGCACACG |
| KRT8 | Forward | AGATGCTGGAGACCAAGTGGAG |
|  | Reverse | GCCGCCTAAGGTTGTTGATGTAG |
| Gapdh | Forward | CAAGGCTGTGGGCAAGGTCATC |
|  | Reverse | TCCAGGCGGCACGTCAGATC |
| Nat10 | Forward | TTCATGGCATCTACTATCAATGGCTAC |
|  | Reverse | CTGGCTGTCGTTGTGGTCTTG |
| ETS2_ac4C | Forward | CTGGAGACGGATGGGAGTTT |
|  | Reverse | CCAGCAAGTTCTGGAGGTCG |
| KRT8_ac4C | Forward | AACAACAAGTTTGCCTCCTTCATAG |
|  | Reverse | CAAATTCGTTCTCCATCTCTGTACG |
| NAT10_prom | Forward | TGCCTTCCTGTGATTTA |
|  | Reverse | AAAGAATGAGCCCTGAG |
| CD274_prom | Forward | GTTCAGATGTTGGCTTGT |
|  | Reverse | GACTTTCCTGACCTTCG |
| siETS2-1 | Forward | CGCCAACUGUGAAUUGCCU |
|  | Reverse | AGGCAAUUCACAGUUGGCG |
| siETS2-2 | Forward | CCUGACUUUGUGGGUGACA |
|  | Reverse | UGUCACCCACAAAGUCAGG |
| siETS2-3 | Forward | CCAACCAUGUCUUUCAAGG |
|  | Reverse | CCUUGAAAGACAUGGUUGG |
| siKRT8-1 | Forward | GCAGCUAUAUGAAGAGGAGAU |
|  | Reverse | AUCUCCUCUUCAUAUAGCUGC |
| siKRT8-2 | Forward | GCCUCCUUCAUAGACAAGGUA |
|  | Reverse | UACCUUGUCUAUGAAGGAGGC |
| siKRT8-3 | Forward | GAGGACUUCAAGAACAAGUAU |
|  | Reverse | AUACUUGUUCUUGAAGUCCUC |
| Oe-KRT8 | KL89792-1-P1 | CTGGACTAGTGGATCCCGCCACCATGTCCATCAGGGTGACCCAGAAGT |
|  | KL89792-1-P2 | TTGTAGTCACTTAAGCTTGGTACCTTGGGCAGGACGTCAGAGGACTCA |
| Oe-ETS2 | 108947-1-p1 | CACACTGGACTAGTGGATCCCGCCACCATGAATGATTTCGGAATCAAG |
|  | 108947-1-p2 | AGTCACTTAAGCTTGGTACCGAGTCCTCCGTGTCGGGCTGGACGC |
| sh-NAT10#1 |  | GCTGTAAGACTCTAGACCAGG |
| sh-NAT10#2 |  | GCCTCAACATCACTCGGATAG |
| sh-NAT10#3 |  | GCTGCTGCAGATGTACTATGA |
| sh-Nat10#1 |  | ATAACCTCCACACGCTGTTTG |
| sh-Nat10#2 |  | TCTCGGAACATGGTCGATTAC |
| sh-Nat10#3 |  | GTATCAGGAGCATCTGGATTA |

**Table S2 The antibodies and reagents used in this study**

| **Antibodies** | **Company** | **Cat. Number** |
| --- | --- | --- |
| NAT10 | Abmart | T510105 |
| ETS2 | Biodragon | RM1905 |
| PD-L1 | Biodragon | RM3478 |
| KRT8 | Abmart | TP51203 |
| N4-acetylcytidine (ac4C) | Abcam | ab252215 |
| IgG | proteinbio | PA2202 |
| CD8α | proteintech | 29896-1-AP |
| GAPDH | proteinbio | PB1034 |
| β-Tubulin Antibody | Abmart | PS16386 |
| FITC CD3 | BD | 553061 |
| R718(AF700) CD8 | BD | 566985 |
| PE-CY7 Granzyme B | biolegend | 372214 |
| Percp-cy5.5 CD4 | BD | 550954 |
| BV510 L/D | BD | 564406 |
