# Supplementary material for: Targeting the ac4C ‘Writer’ NAT10 enhances pancreatic cancer immunotherapy via dual modulation of CD8+ T cells and tumor cells
Source: Cell Death Dis. 2025 Nov 7;16(1):809. doi: 10.1038/s41419-025-08156-0 (PMC12594761; doi:10.1038/s41419-025-08156-0)
Supplement: Supplementary file 2 — supplementary figure [file 41419_2025_8156_MOESM2_ESM.docx]

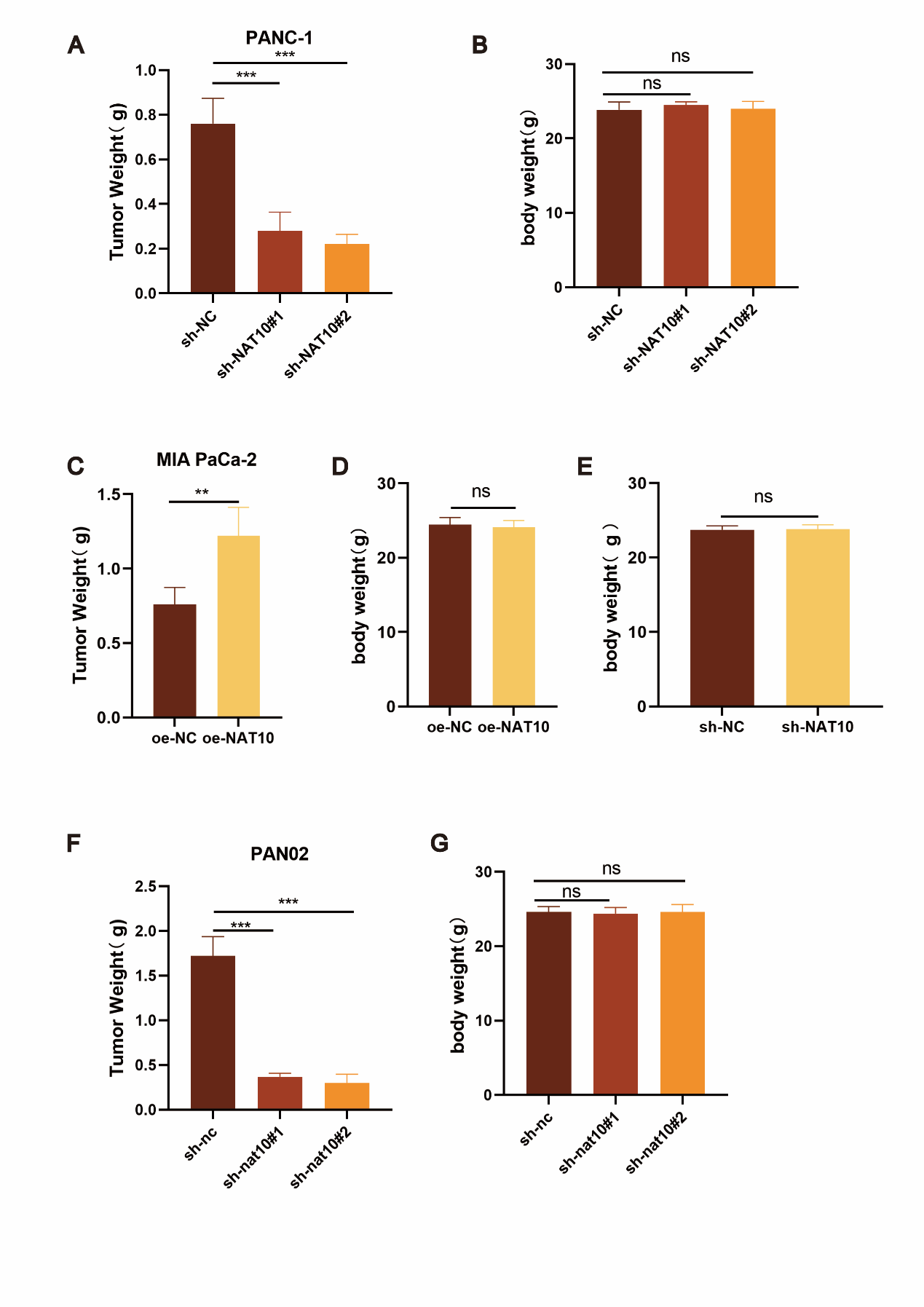


**Figure S1.Animal body weight and tumor mass.A.** Subcutaneous tumor weight in nude mice with NAT10 knockdown versus control group (*P*< 0.001). **B.** Body weight of nude mice with subcutaneous tumor formation in NAT10 knockdown versus control group. **C.** Subcutaneous tumor weight in nude mice with NAT10 overexpression versus control group (*P*< 0.005). **D.** Body weight of nude mice with subcutaneous tumor formation in NAT10 overexpression versus control group. **E.** Body weight of nude mice in lung metastasis model with NAT10 knockdown versus control group. **F.** Subcutaneous tumor weight in mice with Nat10 knockdown versus control group (*P*< 0.001). **G.** Body weight of mice with subcutaneous tumor formation in Nat10 knockdown versus control group.


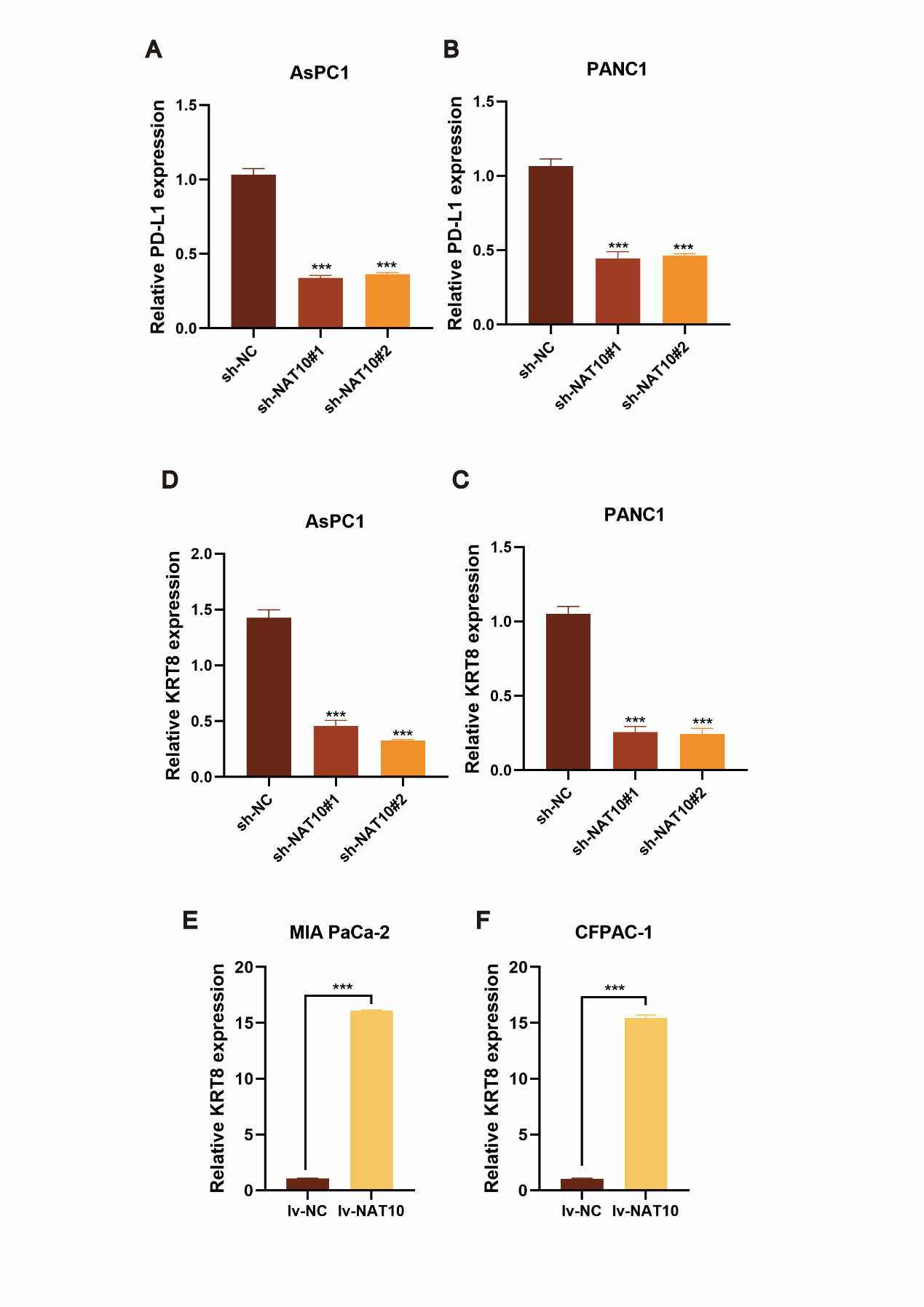


**Figure S2.Expression levels of downstream gene RNAs following knockdown and overexpression of NAT10.A-B.**NAT10 knockdown significantly reduces PD-L1 mRNA expression in PANC-1 and AsPC-1 cells (*P*< 0.001). **C-D.** KRT8 knockdown significantly reduces its mRNA expression in PANC-1 and AsPC-1 cells (*P*< 0.001). **E-F.** KRT8 overexpression significantly increases its mRNA expression in CFPAC-1 and MIA PaCa-2 cells (*P*< 0.001).


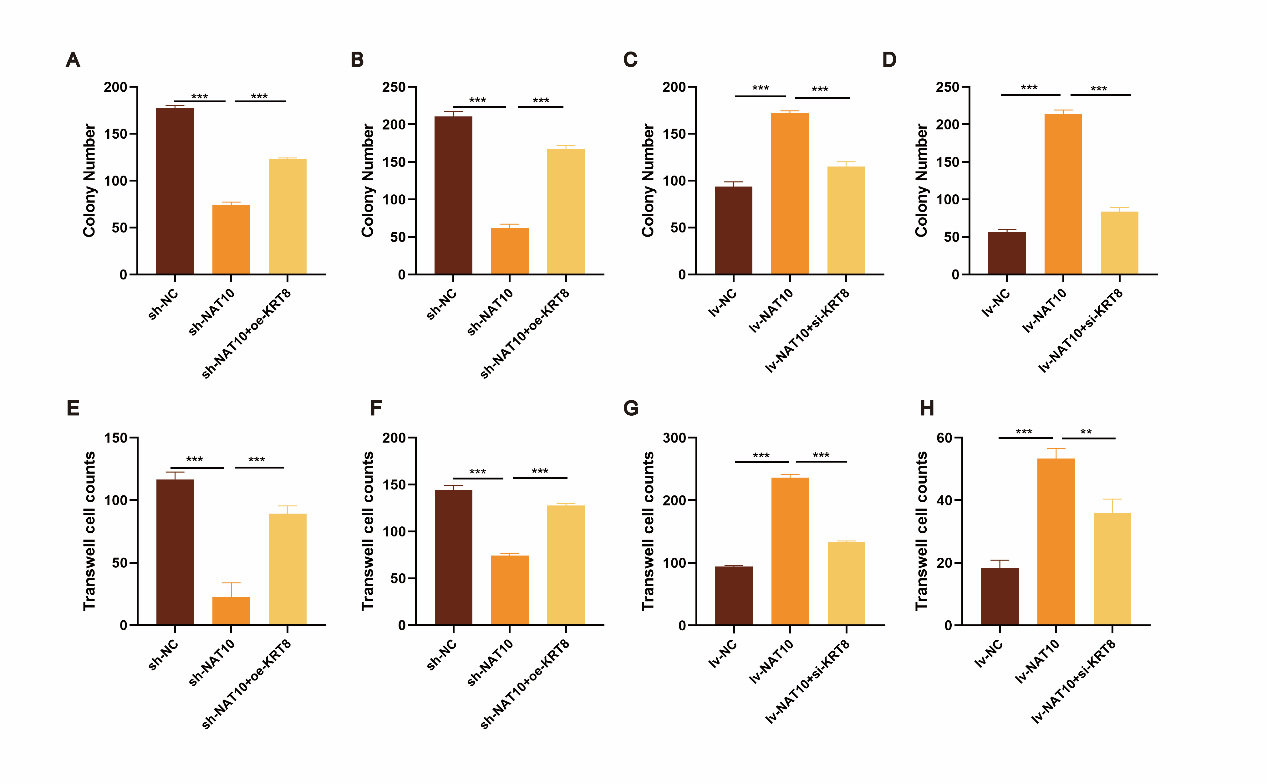


**Fig S3.Quantitative graphs of Colony and Transwell recovery assays.A.** Colony formation assays revealed that NAT10 knockdown reduces colony formation in AsPC-1 cells and that KRT8 overexpression rescues this effect (*P*< 0.001).**B.** Colony formation assays revealed that NAT10 knockdown reduces colony formation in PANC-1 cells and that KRT8 overexpression rescues this effect (*P*< 0.001).**C.** The colony formation assay demonstrated that NAT10 overexpression increased colony formation in MIA PaCa-2 cells, while KRT8 knockdown rescued this effect (*P*< 0.001).**D.** The colony formation assay demonstrated that NAT10 overexpression increased colony formation in CFPAC-1 cells, while KRT8 knockdown rescued this effect (*P*< 0.001).**E.** Transwell assays revealed that NAT10 knockdown inhibits the migration of AsPC-1 cells, whereas KRT8 overexpression restores migratory capacity (*P*< 0.001).**F.** Transwell assays revealed that NAT10 knockdown inhibits the migration of AsPC-1 and PANC-1 cells, whereas KRT8 overexpression restores migratory capacity (*P*< 0.001).**G.** The Transwell assay revealed that NAT10 overexpression enhanced the migration of MIA PaCa-2 cells, whereas KRT8 knockdown suppressed the migratory capacity (*P*< 0.001). **H.** The Transwell assay revealed that NAT10 overexpression enhanced the migration of CFPAC-1 cells, whereas KRT8 knockdown suppressed the migratory capacity (*P*< 0.005).


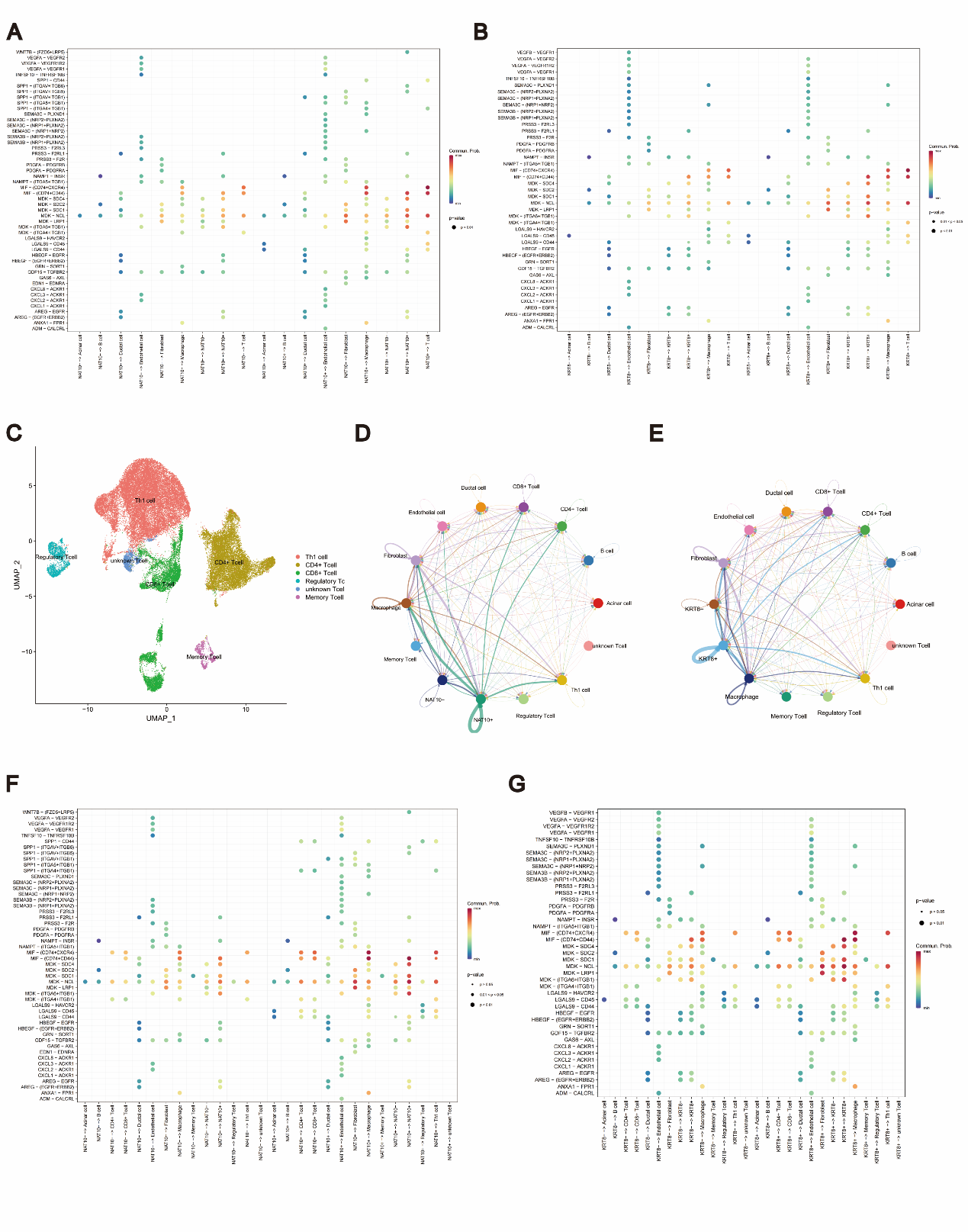


**Figure S4. Single-cell data analysis of ligand-receptor interactions among pancreatic cancer cells. A.** The intercellular ligand-receptor bubble plot illustrates the binding interactions of pancreatic cancer epithelial cells with high expression of NAT10. **B.** The intercellular ligand-receptor bubble plot illustrates the binding interactions of pancreatic cancer epithelial cells with high expression of KRT8. **C.**The UMAP plot demonstrates the distribution of various T cell subsets.**D.** The cell-cell communication plot illustrates the interactions between epithelial cells with high and low expression of NAT10 and different cells.**E.** The cell-cell communication plot illustrates the interactions between epithelial cells with high and low expression of KRT8 and different cells.**F.** The intercellular ligand-receptor bubble plot illustrates the binding interactions of pancreatic cancer epithelial cells with high expression of NAT10. **G.** The intercellular ligand-receptor bubble plot illustrates the binding interactions of pancreatic cancer epithelial cells with high expression of KRT8.


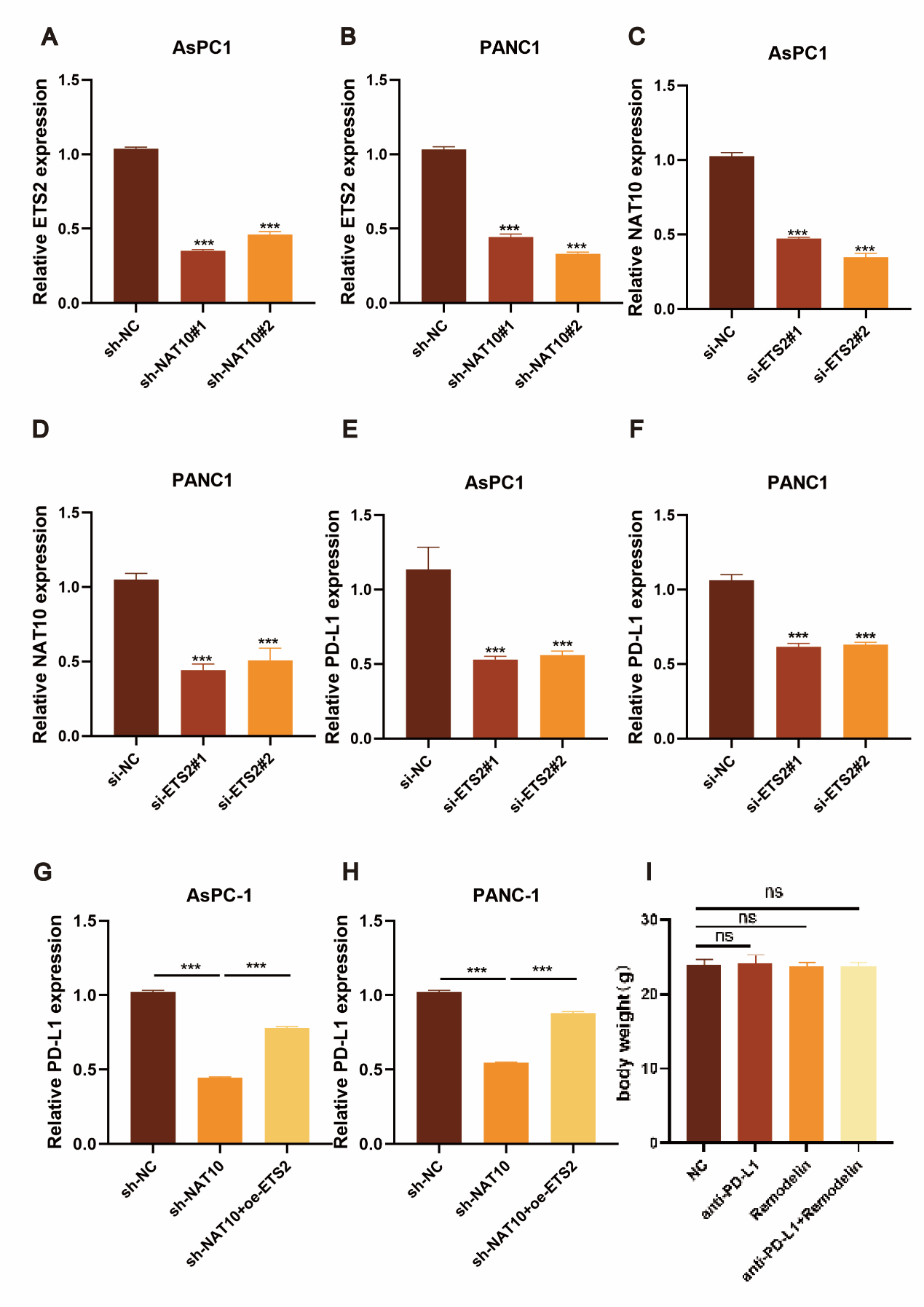


**Fig S5. qPCR results show that NAT10 promotes PD-L1 expression through ETS2.A‒B.** NAT10 knockdown significantly decreases ETS2 mRNA expression in PANC-1 and AsPC-1 cells (*P*< 0.001).**C‒D.** ETS2 knockdown significantly reduces NAT10 mRNA expression in PANC-1 and AsPC-1 cells (*P*< 0.001).**E‒F.** ETS2 knockdown significantly decreases PD-L1 mRNA expression in PANC-1 and AsPC-1 cells (*P*< 0.001).**G-H.** The qPCR results showed that after knocking down NAT10 and overexpressing ETS2, PD-L1 expression was restored (*P*< 0.001).**I.** Body weights of each group in the combined treatment mouse model (*P*< 0.001).
